# Deciphering craniopharyngioma subtypes: Single-cell analysis of tumor microenvironment and immune networks

**DOI:** 10.1016/j.isci.2024.111068

**Published:** 2024-10-01

**Authors:** Tatsuma Matsuda, Takashi Kono, Yuki Taki, Ikki Sakuma, Masanori Fujimoto, Naoko Hashimoto, Eiryo Kawakami, Noriaki Fukuhara, Hiroshi Nishioka, Naoko Inoshita, Shozo Yamada, Yasuhiro Nakamura, Kentaro Horiguchi, Takashi Miki, Yoshinori Higuchi, Tomoaki Tanaka

**Affiliations:** 1 Department of Neurological Surgery Chiba University Graduate School of Medicine, Chiba, Japan; 2 Department of Molecular Diagnosis, Graduate School of Medicine, Chiba University, Chiba, Japan; 3 Research Institute of Disaster Medicine, Chiba University, Chiba, Japan; 4 Department of Aritificial Intelligence Medicine, Graduate School of Medicine, Chiba University, Chiba, Japan; 5 Department of Hypothalamic and Pituitary Surgery, Toranomon Hospital, Tokyo, Japan; 6 Hypothalamic and Pituitary Center, Moriyama Memorial Hospital, Tokyo, Japan; 7 Division of Pathology, Faculty of Medicine, Tohoku Medical and Pharmaceutical University, Miyagi, Japan; 8 Department of Medical Physiology, Graduate School of Medicine, Chiba University, Chiba, Japan

**Keywords:** Cancer, Transcriptomics

## Abstract

Craniopharyngiomas, including adamantinomatous (ACP) and squamous papillary (PCP) types, are challenging to treat because of their proximity to crucial pituitary structures. This study aimed to characterize the cellular composition, tumor tissue diversity, and cell-cell interactions in ACPs and PCPs using single-cell RNA sequencing. Single-cell clustering revealed diverse cell types, further classified into developing epithelial, calcification, and immune response for ACP and developing epithelial, cell cycle, and immune response for PCP, based on gene expression patterns. Subclustering revealed the enrichment of classical M1 and M2 macrophages in ACP and PCP, respectively, with high expression of pro-inflammatory markers in classical M1 macrophages. The classical M1 and M2 macrophage ratio significantly correlated with the occurrence of diabetes insipidus and panhypopituitarism. Cell-cell interactions, particularly involving CD44-SPP, were identified between tumor cells. Thus, we developed a comprehensive cell atlas that elucidated the molecular characteristics and immune cell inter-networking in ACP and PCP tumor microenvironments.

## Highlights

• Cellular composition and cell-cell interactions in ACP and PCP were studied
• Classical M1 and M2 macrophages were enriched in ACP and PCP, respectively
• M1:M2 was linked with diabetes insipidus and panhypopituitarism
• Cell-cell interactions, involving CD44-SPP, were identified in tumor cells

## Graphical abstract

**Figure:**
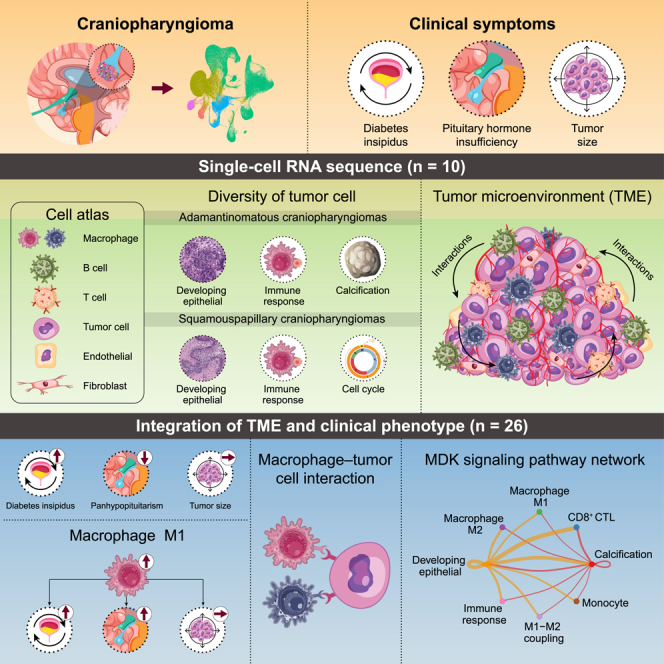

## Introduction

Craniopharyngioma is a rare embryonal malformation of the sella and parasellar region with low histologic malignancy.^1^ With an incidence of 0.5–2 cases/million individuals each year, 30–50% cases occur during childhood and adolescence.^1^ Surgery is the primary treatment for adamantinomatous craniopharyngioma (ACP). However, the aggressive nature of the tumor, along with its anatomical location between the pituitary stalk, optic chiasm, and particularly the hypothalamus, pose challenges for gross total resection. As the tumor grows, it adheres to and infiltrates structures in the mesencephalic and pituitary regions, causing severe neurological deficits. Therefore, surgery alone cannot address the clinical issues associated with craniopharyngiomas. Precision treatments are essential for effective tumor resection while minimizing damage to surrounding healthy structures; therefore, the biological features and molecular mechanisms underlying craniopharyngioma development and tumorigenesis must be comprehensively understood.

Craniopharyngiomas are classified into ACP and squamous papillary (PCP) types. ACP is primarily associated with mutations in exon 3 of *CTNNB1*,^2^ whereas PCP is linked to mutations in *BRAF*.^3^ However, the molecular biology of both types remains incompletely characterized. Yu et al. performed single-cell RNA sequencing (scRNA-seq) of ACP and reported that the reactive glial scar, which formed during craniopharyngioma invasion, established a unique tumor microenvironment (TME) containing diverse immune and glial cells.^4^ TME plays a crucial role in shaping the local tissue microenvironment, which promotes growth in various tumors.^5^ Various immune and stromal cells, including fibroblasts and vascular endothelial cells, contribute to its proliferation and progression within the TME, through interactions with cancer cells, particularly tumor-associated macrophages (TAM). TAMs, comprising classical M1 and M2 macrophages and an intermediate M1-M2 coupling mode^6^ play a significant role in tissue growth and immune responses. However, the role of TAMs in the TME of PCP remains unexplored.

Craniopharyngiomas exhibit high intratumoral diversity^7^; hence, identifying the molecular biology and crosstalk between specific cell types using conventional bulk transcriptomics data is challenging. The advent of scRNA-seq technology allows detailed examinations of intratumoral heterogeneity and the cellular composition of the TME at single-cell resolution.^8^ Therefore, molecular characteristics based on known genetic variants such as *CTNNB1* and *BRAF*^9^ can be identified, and the mechanisms via which cell-cell interactions in the TME influence tumor biology and disease progression can be understood.

This study aimed to characterize the cellular composition, tumor tissue diversity, and cell–cell interactions in enamel epithelial and papillary type craniopharyngiomas samples from 10 patients of various ages, using scRNA-seq. The heterogeneity and intercellular phase-microenvironment interactions were examined in detail and integrated with tumor characteristics and clinical information. We also focused on immune cells, particularly macrophages, within the tumor tissue to explore the intercellular reciprocal network and discuss the molecular biological characteristics and their clinical significance.

## Results

### Clinical characteristics and overall single-cell clustering of 10 craniopharyngioma cases revealed diverse cell types

**Figure 1:**
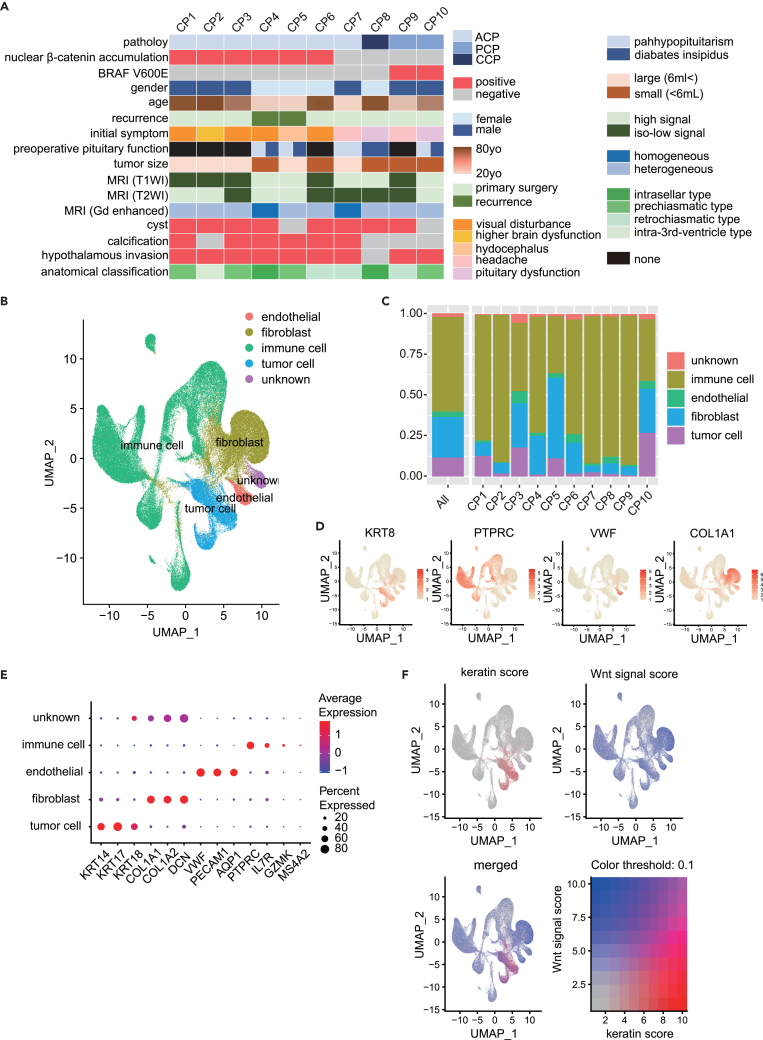
Patient clinical information and cell annotation (A) Among the 10 patients with craniopharyngioma, seven had adamantinomatous craniopharyngioma (ACP), two had squamous papillary craniopharyngioma (PCP), and one had ciliated craniopharyngioma (CCP). (B) Approximately 192,520 cells were clustered using the first 15 principal components (PC15) as determined using the ElbowPlot results, with a 0.1 resolution setting. (C) Histogram depicting the overall and individual case-specific cellular tumor composition ratios. (D) Feature plot of representative marker genes used for cell type annotation: *KRT8* for tumor cells, *PTPRC* for immune cells, *VWF* for endothelial cells, and *COL1A1* for fibroblasts. (E) Dot plot of representative marker genes used for cell type annotation: *KRT14*, *KRT17*, and *KRT18* for tumor cells; *COL1A1*, *COL1A2*, and *DCN* for fibroblasts; *VWF*, *PECAM1*, and *AQP1* for endothelial cells; *PTPRC*, *IL7R*, *GZMK*, and *MS4A1* for immune cells. (F) Feature plot displaying the scores added based on gene sets for keratin and Wnt signaling pathway-related genes. Each score was assigned using the AddModuleScore function of the *Seurat* R package.

The 10 patients included in this study included six men (60%) and four women (40%), aged between 23 and 75 years. Two patients (20%) presented with recurrent tumors, whereas the remaining eight patients (80%) underwent primary surgery. The most common initial symptoms were visual disturbance (four patients, 40%), pituitary dysfunction (two patients, 20%), headache (two patients, 20%), increased brain dysfunction (one patient, 10%), and hydrocephalus (one patient, 10%). Table S1 presents the patient characteristics in detail. Pathological diagnosis helped identify seven cases of ACP (CP1–7), two cases of PCP (CP9–10), and one case of ciliated-type craniopharyngioma (CCP) (CP8) (Figure 1A). CP1 exhibited β-catenin nuclear transfer, and BRAF V600E was identified in CP 9–10. Magnetic resonance imaging results, preoperative anterior pituitary function, central enuresis presence, tumor calcification, hypothalamic invasion, and anatomic classification are detailed in Figure 1A.

To identify cell types within the tumor, the merged data were clustered using up to 15 principal components (PC15) with reference to ElbowPlot results and a resolution of 0.1. Single-cell clusters were classified into 10 clusters (Figures 1B, S1A, and S1B). The number of cells varied in each case; however, cells from each case were distributed across all clusters and were unaffected by batch effects (Figure S1C). Immune cells were identified in clusters 0, 2, 5, 6, and 9 using *PTPRC* (*CD45*), a common leukocyte antigen, *IL7R*, and *MS4A2*, a B-cell marker. Clusters 1 and 4 represented fibroblasts based on *COL1A1* and *DCN* expression, whereas cluster 7 represented vascular endothelial cells based on the expressions of *VWF*, *PLVAP*, and *PECAM1*. Cluster 3, adjacent to the fibroblast cluster, was identified to include tumor cells based on the high expression of keratin-related genes (e.g., *KRT18*) (Figures 1B, 1D, and S1D). Cluster 8 was labeled unknown (Figures 1E and S1F) owing to potential contamination with aberrant genes or doublet cells (Figures 1B, 1D, and S1D). Quality control (QC) results for each identified cell type revealed that “unknown” had the same parameters as other clusters, suggesting that this cell population could be identified in the same way as other cell populations (Figure S1E).

Cell type identification among the cases revealed that approximately 50% tumor tissue cells were immune cells and approximately 10–20% were tumor cells, with varying percentages of fibroblasts (Figure 1C).

As tumorigenesis of ACP is associated with activation of the WNT/β-catenin pathway by *CTNNB1* mutations, we examined the Wnt signaling activity throughout the tumor tissue and found moderately increased activity in tumor cells. Consistent with tumor characteristics, keratin-related genes were specifically upregulated in the tumor cell population, as determined by keratinization scores (Figure 1F).

### Tumor cells are further subclustered into three types based on their gene expression profiles

**Figure 2:**
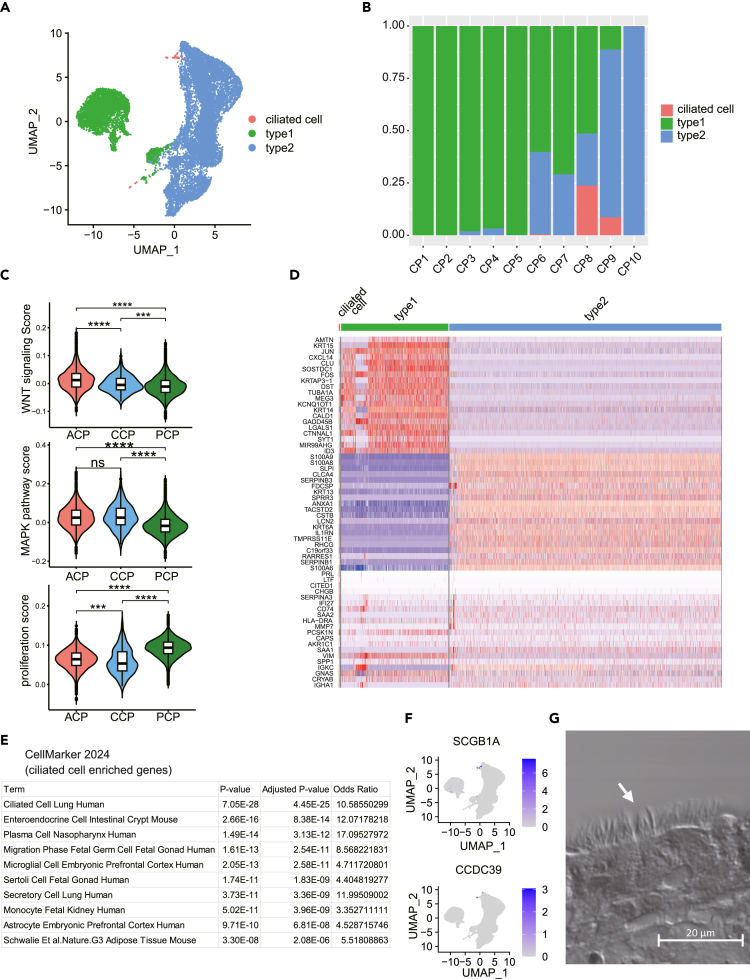
Subclustering of tumor cells (A) Tumor cells were predominantly segregated into type 1 and type 2 tumor cells, with a small cluster of ciliated cells observed. (B) Histogram depicting the composition ratio of tumor cell subtypes for each case. (C) Violin plots representing the scores assigned based on gene sets related to the Wnt signaling pathway, MAPK pathway, and cell proliferation. Each score was assigned using the AddModuleScore function of the *Seurat* R package. The accompanying boxplot shows the interquartile range, and the line inside represents the median.(∗∗∗∗: *p* ≤ 0.0001, ∗∗∗: *p* ≤ 0.001, ∗∗: *p* ≤ 0.01, ∗: *p* ≤ 0.1, ns: not significant). (D) Heatmap of gene expression for the top 10 genes based on log_2_FC among differentially expressed genes (DEGs) in each tumor cell cluster and for genes universally expressed across all tumor cell clusters. DEGs were identified using a threshold of Log_2_FC > 0.25 and expression >25% of cells of each type, using the FindAllMarkers function of the *Seurat* package. (E) Cell type annotation of the ciliated cell cluster based on DEGs (Descartes Cell Type and Tissue 2021^10^). (F) Featureplots for the expression of ciliated cell-associated genes. (G) Histological image of CP8 (ciliated craniopharyngioma: CCP) indicating cilia (white arrow). Scarl bar, 20μm.

To reveal the diversity of the 19,290 tumor cells, clustering was performed at a cluster resolution of 0.5 (Figure S2A) and cell proportions of the 31 clusters were pathologically diagnosed into ACP, PCP, and CCP (Figure S2B). Clusters of only ACP- and PCP-derived cells and mixed cell clusters were found; therefore, tumor cells were subclustered into three categories (Figure 2A). The variable gene expression profiles of clusters, particularly in a small cell subset, were identified via enriching genes, such as *CCDC153*, *CCDC181*, *CCDC17*, and *CCDC39*, which were predominantly expressed in ciliated epithelial cells, as previously reported^7^ (Figures 2D and 2E), suggesting their association with ciliated cells (Figure 2F). Consequently, we categorized the three cell groups as type 1 and type 2 ciliated cells (Figure 2A).

The tumor cells, excluding the ciliated cells, formed two major clusters, representing the primary cell types of craniopharyngioma (Figures 2A and 2B). Type 1 and type 2 tumor cells constituted 28% and 72% of all tumor cells, respectively. In terms of the tumor cell composition per patient, CP1/2/3/4/5 predominantly featured type 1 tumor cells, CP9/10 mainly exhibited type 2 tumor cells, and CP6/7/8 exhibited a mixture of both (Figure 2B). Distinctions among the type 1 tumor, type 2 tumor, and ciliated cell groups suggested diverse gene expression backgrounds beyond those recognized as differentially expressed genes (DEGs) (Figure 2D; Table S3).

For each tissue—ACP, PCP, and CCP—the Wnt signal-related genes, MAPK pathway-related genes activated by BRAF V600E,^11^^,^^12^ and genes related to cell proliferation were scored using the AddModuleScore function. The Wnt signaling and MAPK pathway scores were remarkably higher in ACP, whereas the cell proliferation score was significantly increased in PCP, compared to those in the other two tissues (Figure 2C).

A small number of ciliated cells were observed in CP8 and the pathological image of CP8 displayed goblet cells with villi (Figure 2G).

### Gene expression and pathology in the classification of type 1 and type 2 cells aid in distinguishing between both types based on *DKK3* and *S100A8* expression patterns

**Figure 3:**
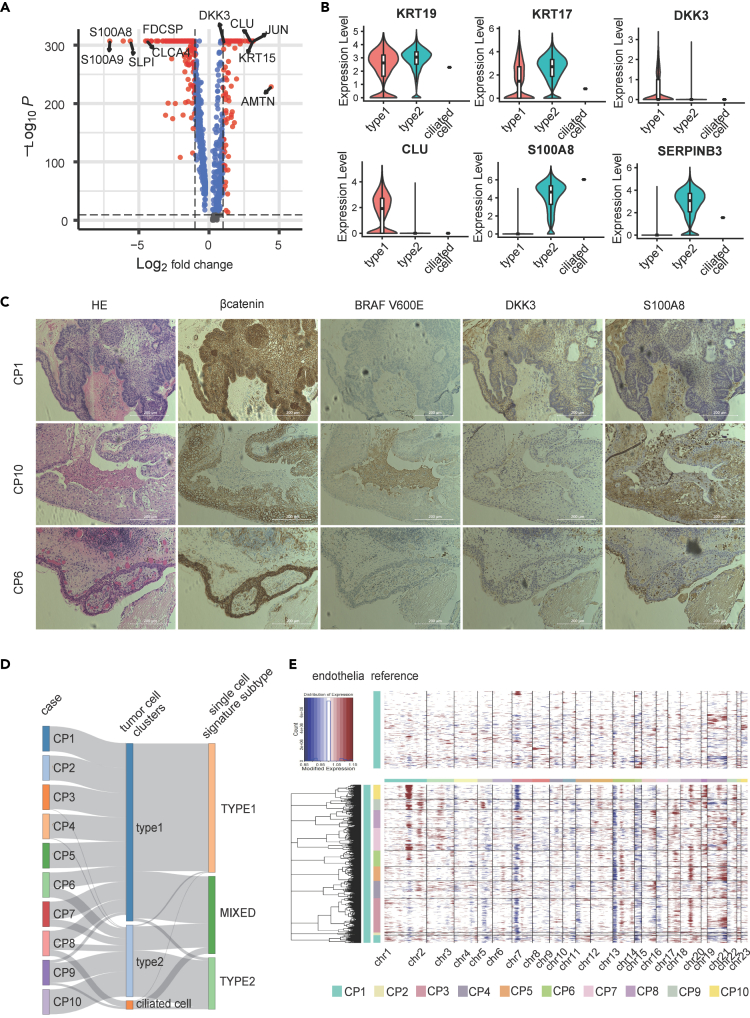
Comparison between type 1 and type 2 tumor cells and diagnostic marker identification (A) Volcano plot displaying DEGs between type 1 and 2 tumor cells. DEGs were identified using the FindAllMarkers function of the *Seurat* R package, with a threshold of Log_2_FC > 0.25 and expression in more than 25% of cells in each type. Genes upregulated in type 1 and type 2 tumor cells are displayed on the right and left, respectively. Genes with Log_2_FC > 1 are highlighted in red. (B) Violin plots depicting the expression comparison of genes highly specific to each tumor cell type. The accompanying boxplot shows the interquartile range, and the line inside represents the median. (C) Immunostaining results for highly specific markers. Scale bar, 200 μm. (D) Sankey plot depicting the distribution of tumor cell types in individual cases, displayed as the percentage of each tumor cell type for each case. (E) Copy number variation (CNV) inferred for each tumor cell using immune cells as a reference, calculated using the *inferCNV* R package. Each row represents a single cell.

Variable expression gene analysis was performed to elucidate the distinct genetic backgrounds of the type 1 and type 2 tumor cell categories. We identified 1,416 DEGs (514 upregulated in type 1 and 902 upregulated in type 2) between the two groups (Figure 3A; Table S4). Gene Ontology (GO) analysis of identified DEGs revealed that type 1 tumor cells were enriched in peptide metabolic (GO:0006518), amide biosynthetic (GO:0043604), cellular nitrogen compound biosynthesis (GO:0044271), amide metabolic (GO:0043603) (Figure S3), and all processes implicated in translation and transcription. Conversely, type 2 tumor cells were enriched in tissue (GO:0009888) and epithelial (GO:0060429) development (Figure S3).

The expression levels of *CLU*/*DKK3* (plasma protein) in type 1, and *S100A8*/*SERPINB3* (plasma protein) and *LCN2* (nuclear and plasma protein) in type 2 DEGs indicate that each gene was valuable for differentiation. Keratins, such as *KRT19* and *KRT17*, were consistently expressed, suggesting their potential role as common markers for craniopharyngioma tumor cells (Figure 3B). In CP1 (ACP), comprising type 1 tumor cells, the nuclear migration of β-catenin was observed in the wheel structure. DKK3, a type 1 tumor cell marker, was localized in the center and surrounding area of the wheel structure (Figure 3C). In contrast, in CP10 (PCP), consisting of type 2 tumor cells, DKK3 staining was weaker than that in CP1, and S100A8, a type 2 tumor cell marker, was observed around the BRAF V600E-stained area. In CP6 (ACP), a mixture of type 1 and type 2 tumor cells, the nuclear translocation of β-catenin and BRAF V600E were not evident. Both DKK3 and S100A8 exhibited weaker staining in CP6 than those in CP1 and CP10 (Figure 3C). Immunostaining of samples from the remaining seven patients yielded similar results (Figure S4). These findings suggest that type 1 DKK3 and type 2 S100A8 are effective for differentiating between the two types.

CP6/7/8 were designated as mixed-type as they contained approximately 50% type 1 or type 2 tumor cells (Figures 2B and 3D). Therefore, we hypothesized that CP6 had a distinct gene expression profile from either type 1 or 2. Hence, we performed single-cell genomic DNA copy number variation (CNV) analysis using inferCNV.^13^ In CP9 and CP10, comprising type 2 tumor cells, gain in the long arm of chromosome 1 was observed in almost all cells, in contrast to type 1. Conversely, compared with type 1 tumor cells, CP6, CP7, and CP8, constituting mixtures of type 1 and type 2 tumor cells, exhibited this gain in only a fraction of tumor cells. This indicates the presence of genomic heterogeneity in CP6/7/8 tumor cells of the mixed-type (Figure 3E).

### Diversity and the molecular basis of ACP tumor cells

**Figure 4:**
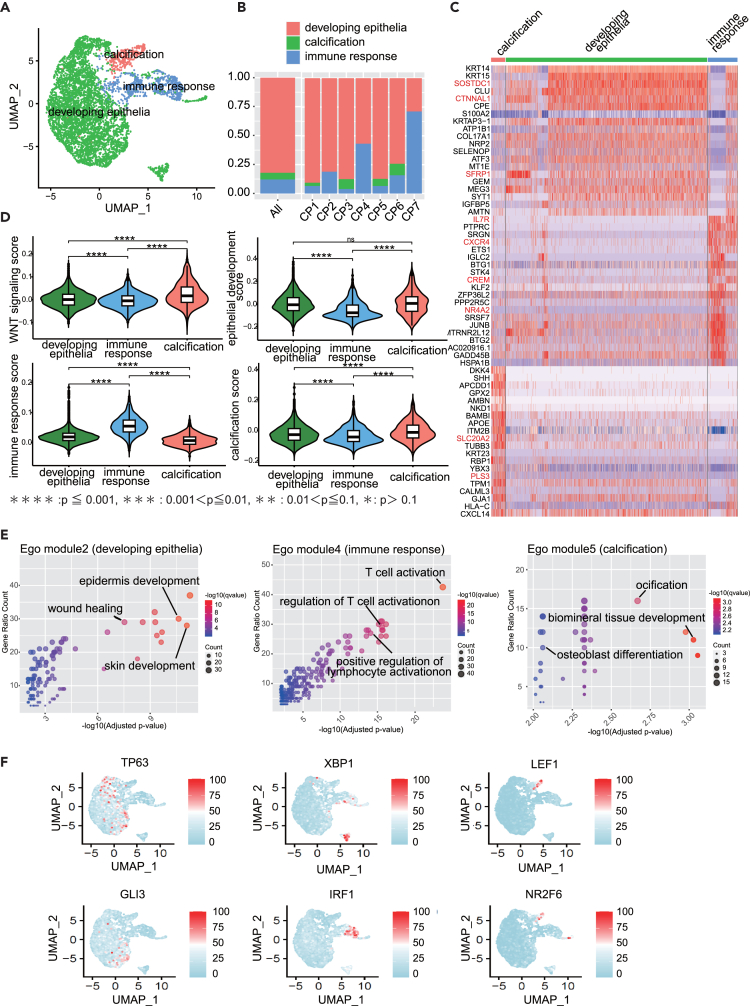
Heterogeneity of adamantinomatous craniopharyngioma (ACP) tumor cells and biological characteristics and transcription factors of each subtype (A) Uniform Manifold Approximation and Projection (UMAP) of ACP tumor cells revealing three distinct tumor subtypes, color-coded by subtype classification. (B) Histogram depicting the composition ratio of ACP tumor cell subtypes, overall and for each case. (C) Heatmap of gene expression for the top 10 genes based on log_2_FC among differentially expressed genes (DEGs) in each ACP tumor cell subtype. DEGs were identified using a threshold of log_2_FC > 0.25 and expression in more than 25% of cells of each type using the FindAllMarkers function of the *Seurat* R package. (D) Violin plots displaying the scores assigned from gene sets of biological processes indicative of each subtype characteristics. Each score was assigned using the AddModuleScore function of the *Seurat* package. The accompanying boxplot shows the interquartile range, and the line inside represents the median. (∗∗∗∗: *p* ≤ 0.0001, ∗∗∗: *p* ≤ 0.001, ∗∗: *p* ≤ 0.01, ∗: *p* ≤ 0.1, ns: not significant). (E) Scatterplot displaying enriched biological process terms for module 2 (developing epithelial), module 4 (immune response), module 5 (calcification). (F) Featureplot depicting the activity of transcription factors specific to each ACP tumor cell subtype, as determined using SCENIC.

We subclustered only ACP tumor cells to comprehensively elucidate their diversity. ACP tumor cells were categorized into three clusters (Figure 4A; Table S5), and their composition varied within the tissue (Figure 4B). DEGs of each subcluster were analyzed, and clusters enriched with epithelial-forming genes, such as *SOSTDC2*,^14^^,^^15^ *CTNNAL1*,^16^ and *SERP1*,^17^ were defined as “developing epithelia.” Similarly, clusters enriched with genes, such as *IL7R*,^18^ *CXCR4*,^19^ and *CREM*,^20^ involved in immune response, were defined as “immune response; ” those with prominent genes, such as *SLC20A2*^21^ and *PLS3*,^22^ involved in calcification, were defined as “calcification” (Figures 4A and 4C).

To confirm the validity of this classification, each subcluster was scored using gene sets, which revealed that the epithelial-developing, immune response, and calcification scores were respectively highest in the relevant groups, indicating a reasonable cluster classification scheme (Figure 4D). The gene expressions of *CTNNB1* and *WNT5A* were evenly distributed among the three clusters, and no expression was specific to any particular cluster (Figure S5A). However, when the gene set involved in the Wnt signal was scored using the AddModuleScore function, the Wnt signal was significantly enhanced in calcification (Figure 4D). Similarly, the expressions of *TOP2A*^23^ and *MKI67*,^24^ involved in cell proliferation, were also confirmed; however, only a few expressing cells were scattered (Figure S5C). *VCAN*^25^ and *CD47*,^26^ involved in calcification and apoptosis, respectively, were not clustered at the single gene level in calcification and immune response, suggesting a complex interplay of multiple genes in the molecular basis that determined the characteristics of these three clusters (Figures 4D and S5C).

To comprehensively characterize the molecular biology of each cluster, the gene module analysis using monocle3^27^ found that developing epithelial cells were strongly enriched in module 5 and 11, associated with “wound healing.” Epithelial development was also strongly enriched in module 11 and contained terms related to epithelial development, such as “wound healing,” “epidermis development,” and “skin development” (Figure 4F). Modules 6,7, and 15 were enriched in calcification (an ACP clinical feature), containing related terms such as “ossification” and “odontogenesis.” Immune responses were weakly enriched in modules 1, 2, 3, 10, 13, 14, and 16; EGO in module 2 included related terms such as “T cell activation,” “leukocyte-mediated immunity,” and “lymphocyte-mediated immunity.” This suggests that the immune response group represents a cell population interacting with immune cells in the tumor tissue (Figures 4E and 4F).

To elucidate the molecular regulatory mechanisms controlling these subcluster characteristics, we searched for activating transcription factors using single-cell regulatory network inference and clustering (SCENIC)^28^ and identified 104 transcription factors (Figure S5D; Table S6). In the developing epithelia, the activities of *TP63*^29^ and *GLI3*^30^ were high, which determined the characteristics of developing epithelial cells, such as “epidermal development.” In calcification, *LEF1*^31^ and *NR2F6*^32^ were more active than *TP63* and *GLI3*. *IRF1*^33^ and *XBP1*,^34^ which are involved in “T cell activation,” were detected as highly active transcription factors in immune response (Figures 4G and S5D; Table S7).

These results suggest that ACP tumor cells subcluster as a cell population in the three distinct subtypes with different molecular biological bases: developing epithelial, calcification, and immune response.

### Diversity and molecular biological basis of PCP tumor cells

**Figure 5:**
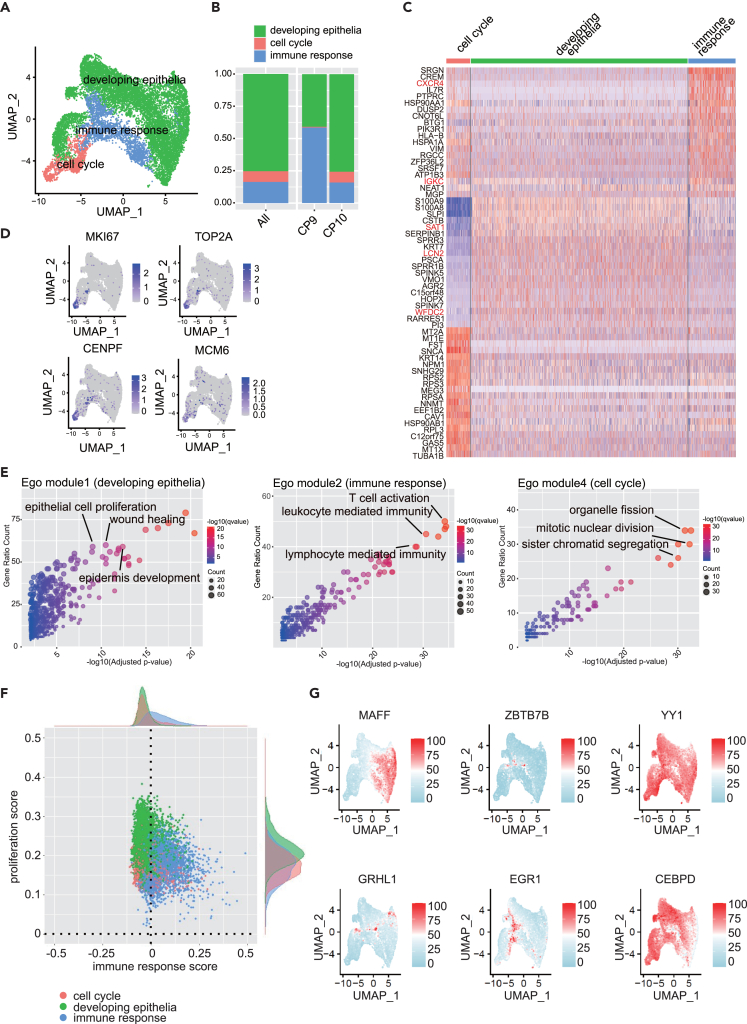
Heterogeneity of papillary craniopharyngioma (PCP) tumor cells and biological characteristics and transcription factors of each subtype (A) Uniform Manifold Approximation and Projection (UMAP) of PCP tumor cells revealing three distinct tumor subtypes, color-coded by subtype classification. (B) Histogram displaying the composition ratio of PCP tumor cell subtypes, overall and for each case. (C) Heatmap of gene expression for the top 10 genes based on log_2_FC among differentially expressed genes (DEGs) in each PCP tumor cell subtype. DEGs were identified using a threshold of Log_2_FC > 0.25 and expression in more than 25% of cells of each type using the FindAllMarkers function of the *Seurat* R package. (D) Featureplot displaying expressions of the proliferation markers *MKI67*, *TOP2A*, *CENPF* and *MCM6*. (E) Scatterplot displaying enriched biological process terms for module 1 (developing epithelia), module 2 (immune response), and module 4 (cell cycle). (F) Scatterplot with the proliferation score on the vertical axis and the immune response score on the horizontal axis. The dot color indicates different tumor cell subtypes in PCP. Each score was assigned using the gene sets for module 2 (immune response) and module 4 (cell cycle) using the AddModuleScore function of the *Seurat* package. (G) Featureplot depicting the activity of transcription factors specific to each PCP tumor cell subtype, as determined using SCENIC.

To comprehensively capture the nuanced tumor cell diversity within PCP, we specifically selected CP9 and CP10 tumor cells for subclustering and categorized them into three clusters (Figures 5A, 5B, S6A, and S6B). The DEGs of each subcluster were identified; clusters enriched with genes, such as *WFDC2*,^35^ *LCN2*,^36^ and *SAT1*,^37^ involved in epithelial formation, were labeled as “developing epithelial.” *WFDC2* was associated with epithelial-mesenchymal transition in tumor tissues. Similarly, clusters enriched with genes involved in immune response, such as *CXCR4* and *IGKC*, were designated as “immune response.” (Figures 5A and 5C; Table S7) Clusters expressing genes, such as *TOP2A*,^23^ *CENPF*^38^*,* and *MKI67*,^24^ involved in cell division, were defined as the cell cycle (Figure 5D). A few cells were also identified in the developing epithelial and immune response subclusters (Figure 5D).

Gene module analysis, performed using Monocle3, elucidated the distinctive characteristics of each subcluster. Modules 2, 3, and 5 were enriched in the immune response, associated with terms such as “T cell activation,” “leukocyte mediated immunity,” and “lymphocyte mediated immunity.” Module 1 was strongly enriched in developing epithelial and played a role in “wound healing” and “epithelial cell proliferation.” Module 4 was enriched in “organelle fission,” “mitotic nuclear division,” and “sister chromatid segregation” (Figures 5E and S6B; Table S7). Comparing the enrichment degree of each module across clusters revealed that the modules of epithelial formation, cell proliferation, and immune response were inversely enriched in immune responses relative to the modules of developing epithelial and the cell cycle (Figure 5E).

Subsequently, using the gene sets contained within each module, the subclusters were scored. The results demonstrated that many cells in the cell cycle exhibited high proliferation and low immune response scores. Conversely, the immune response cluster displayed low proliferation and high immune response scores, whereas the developing epithelial cluster exhibited an intermediate profile between the two (Figure 5F).

To delineate the molecular basis governing these subcluster characteristics, we searched for transcription factors using SCENIC and identified 88 transcription factors (Figure S6C; Table S9). Among these, *SMARCA4*^39^ was identified, which was involved in holding sister chromatids together in the cell cycle (Figure S6C). *CEBPB*,^40^ a transcription factor that regulated cell proliferation genes, displayed high activity in the developing epithelia and was included in “epithelial cell proliferation.” *MAFF*,^41^ *GRHL1*,^42^ and *FOXC1*,^43^ associated with epithelial formation, including “epidermis development,” were also identified (Figure S6C). *ZBTB7B*^44^ and *EGR1*^45^ were highly active transcription factors in immune response, involved in “T cell activation” (Figures 5G and S6C). Overall, the identified transcription factors in each subcluster of the PCP tumor cells exhibited specific activation in their respective clusters (Figure 5G). Collectively, the subcluster classification of developing epithelial, cell cycle, and immune response as subtypes with three distinct molecular biological bases in PCP tumor cells appears justified.

### Immune cell subclustering and its impact on the clinical characteristics of macrophage subtypes

To elucidate the role of immune cells in combating tumor cells and their influence on the TME, subclustering was performed on 96,746 immune cells, using principal components up to PC10 based on ElbowPlot results and a cluster resolution of 0.7. A total of 19 clusters were identified (Figure S7C). Each cluster included T cell markers (*CD3G*, *CD3D*, *CD3E*, *CD8B*, *CD8A*, *CXCR3*, *GZMK*, *NR4A2*, *CD69*, *IL7R*, KLF2, *TCF7*, *EF1*, *CCR7*, *SELL*), cytotoxic markers (*GNLY*, *KLRF1 KLRD1*, *GZMB*, *ZNF683*, *NKG7*, *PRF1*), B-cell markers (*MS4A1*, *CD79A*), plasma-like B-cell markers (*FKBP11*, *DERL*, *IGHA1*, *IGHG3*), mast cell markers (*KIT*, *GATA2*), and myeloid markers (HLA-DRB5, HLA-DRB1, HLA-DRA, *RAMP1*, *CCL22*, *LAD1*, *IDO1*, *GPNMB*, *FCAR*, *C1QC*, *C1QA*, *RNASE1*, *S100A9*, *S100A8*, *VCAN*, *CD14*), which were used for annotation (Figures S7A and S7D).

While the proportion of immune cell types varied among cases, myeloid cells constituted the largest portion overall, accounting for approximately 40% (Figure S7B). These 39,185 myeloid cells were subclustered and identified by *CD14*, *VCAN*, *S100A8*, *RNASE1* monocyte/macrophage markers, and *CCR7*, *IL3RA*, *IDO1* DC markers (Figures S7E–S7G).

**Figure 6:**
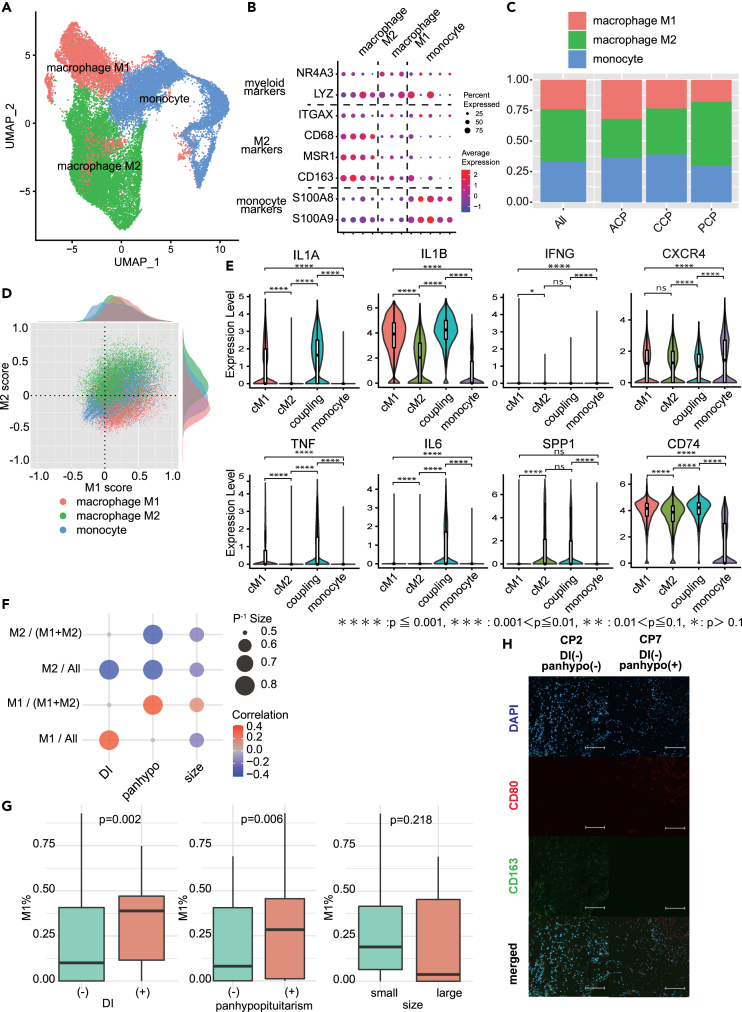
Subtyping of tumor-associated macrophages (TAMs) and their impact on clinical symptoms (A) Uniform Manifold Approximation and Projection (UMAP) of myeloid cell color-coded by subtype (monocyte/macrophage M1/macrophage M2). (B) The dot plot displays the expression of marker genes common to myeloid cells and those specific to subtypes. (C) Histogram displaying the composition ratio of myeloid subtypes by type. (D) Scatterplot with M1 and M2 scores on the horizontal and vertical axes, respectively. Dot color corresponds to the myeloid subtypes presented in Figure 6A. Graph quadrants are defined as classical M1, classical M2, M1-M2 coupling, and monocyte. Each score was assigned using the AddModuleScore function of the *Seurat* R package. (E) Violin plot displaying the expressions of immune-related genes. (∗∗∗∗: *p* ≤ 0.0001, ∗∗∗: *p* ≤ 0.001, ∗∗: *p* ≤ 0.01, ∗: *p* ≤ 0.1, ns: not significant, cM1: classical M1, cM2: classical M2, coupling: M1-M2 coupling. (F) Dot plot illustrating the impact of calculated macrophage type (CMT) ratios on clinical phenotypes. Ratios are categorized as high or low; both correlation coefficients and *p*-values were calculated. The correlation coefficients were determined using Pearson’s method. cM1: classical M1, cM2: classical M2. (G) Boxplot depicting the percentage of M1 in 10 single-cell cases and an additional 16 cases assessed through immunohistochemistry, categorized by clinical phenotype. The accompanying boxplot shows the interquartile range, and the line inside represents the median. Each case was photographed in 10 fields of view, excluding those with numerous artifacts or obvious staining defects. Eight fields of view were selected per case for counting positive cells. (H) Immunostaining images of macrophage markers. The scale bar is 100 μm.

Subsequently, macrophages/monocytes were subclustered and classified into M1 and M2 macrophages, and monocytes using the macrophage and monocyte markers (Figures 6A and 6B). The proportion of each annotated macrophage subtype in type 1 and 2 mixed tumors was examined. In type 1, M1 and M2 each constituted approximately 50% of the macrophages, whereas the percentage of M2 was higher than that of M1 in type 2 tumor cells (Figure 6C).

Further, macrophage diversity was assessed using AddModuleScore for M1 and M2 macrophages and monocytes, with M1 and M2 scores appended. The results indicated that macrophages annotated with the marker gene had high scores; however, M1 and M2 scores were elevated and correlated. This suggests that many cells exhibited M1 and M2 characteristics in addition to the classical macrophage classification annotated with the marker gene, indicative of the M1-M2 coupling mode^6^ (Figure 6D). Based on these results, cells annotated as monocytes in the marker gene had low M1 and M2 scores and were classified as “calculated macrophage type (CMT)” based on the scoring scatter. Other annotations included “classical M1,” “classical M2,” “M1–M2 coupling,” and “monocyte.” The expressions of *IL1A, IL1B, INFG, TNF*, and *IL6* were significantly higher in “classical M1” than those in “classical M2” (Figure 6E). The percentage of CMT displayed wide case- and clinical type-based diversities (Figures S8A–S8C). We hypothesized that the proportion of macrophage subtypes in the tissue could have influenced the extent of the immune and inflammatory response in the TME. To identify classical M1 carefully and distinguish M2 from monocytes and coupling modes, we set each score to above 0.2 and examined the correlation between the CMT proportion and clinical findings. The results showed that the M1 percentage in all macrophages (classical M1 + classical M2) showed a significant positive correlation with the development of panhypopituitarism (Figures 6F and 6G; Table S2), whereas the M2 percentage showed a negative correlation. (Figures 6F and 6G). To validate these findings, the proportion of classical macrophages was examined via co-staining for M1 (CD80) and M2 (CD163) markers in a previous craniopharyngioma specimen, which revealed similar consistent results (Figure 6H).

### Fibroblast diversity

To explore the molecular characteristics of fibroblast cells within the TME, subclustering was performed and their genetic characteristics were investigated. A total of 55,209 fibroblast cells were divided into two clusters using principal components up to PC15 and a resolution of 0.5 (Figure S9A). While the proportion of fibroblast subtypes between type 1 and type 2 cells showed no notable differences, type 2 fibroblasts were more prevalent than type 1 fibroblasts in mixed-type cells (Figures S9B). A total of 752 DEGs were identified in the fibroblasts, with 590 in type 1 and 162 in type 2 fibroblasts (Figures S9C).

*S100A9, SPP1*, and *S100A8* were detected in type 1 fibroblasts in the detected DEGs (Figure S9D). GO analysis showed the negative regulation of the apoptotic process (GO:0043066), apoptosis process (GO:0042981), programmed cell death (GO:0043069), and characteristics associated with leukocyte aggregation (GO:0070486) (Figure S9E). Conversely, type 2 fibroblasts were enriched in processes related to extracellular matrix (GO:0030198) and supramolecular fiber (GO:0097435) organization; *MGP, SRFP2*, and *SFRP4* were identified as DEGs and contributed to extracellular skeleton formation (Figures S9D and S9E).

### Vascular endothelial cell diversity

Vascular endothelial cells are integral TME components; their molecular characterization revealed 7,123 vascular endothelial cells, which were categorized into 19 clusters using principal components up to PC15 and a resolution of 0.9 (Figures S9F and S9G). All clusters expressed common endothelial (*VWF*/*PVVAP*/*PECAM1*), arterial (*EFNB2*/*HEY1*/*FBLN5*), vein (*NR2F2*/*EPHB4*), capillary (*VCAM1*/*RGCC*), lymphatic (*PROX1*/*LYVE1*), and proliferative (*STMN1*/*MKI67*) markers (Figure S9G). Most endothelial cells in the tumor tissues were capillary endothelial cells; only a few cells exhibited proliferation (Figure S9H).

### Tumor and immune cells in craniopharyngioma participate in cell-cell interactions

**Figure 7:**
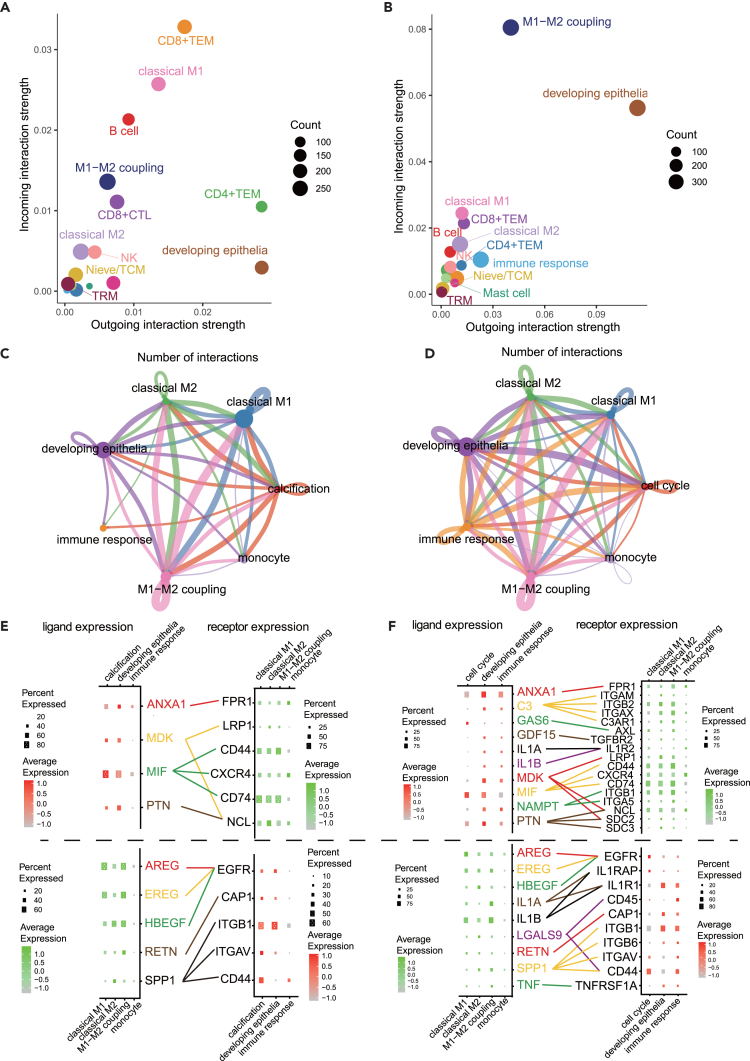
Complex cellular interactions within the tumor microenvironment of craniopharyngiomas Role of cell-cell interactions (sender and receiver) among different cell types in (A), adamantinomatous craniopharyngioma (ACP) and (B), papillary craniopharyngioma (PCP). (C) Interactions between tumor cells and macrophages in ACP. The size of the dot representing each cell type indicates the number of cells. The thickness of the line connecting the dots indicates the signal magnitude. (D) Interactions between tumor cells and macrophages in PCP. The size of the dot representing each cell type indicates the number of cells, and the thickness of the line connecting the dots indicates the signal magnitude. (E) Dot plot and interactions displaying the expression of receptors/ligands among different cell types in ACP. The upper section displays signals sent from each tumor cell subtype to each calculated macrophage type (CMT) type. The lower section denotes signals sent from each CMT type to each tumor cell subtype. Lines connecting signatures represent signals. (F) Dot plot and interactions depicting the expression of receptors/ligands among different cell types in ACP. The upper section shows signals sent from each tumor cell subtype to each CMT type. Lower section shows signals sent from each CMT type to each tumor cell subtype. Lines connecting signatures represent signals.

Cell-cell interactions were analyzed using CellChat to elucidate the role of ACP and PCP in each cell type tissues. The interaction strength of ACP and PCP as signal senders and targets revealed that macrophages, such as the M1–M2 coupling type and classical M1, played crucial roles in both cell types (Figures 7A and 7B).

The interaction between macrophage subtypes and cells in tumor tissues revealed that macrophages were interconnected with other immune and tumor cells, potentially acting as hubs in tumor tissues (Figures 7C and 7D). Hence, we focused on cell-cell interactions between each tumor cell and macrophage subtype.

Initially, signals from tumor cells to various macrophages in ACP tumor tissues were expressed by ligands for *ANXA1* and *MIF* in tumor cell subtypes, defined as calcification. Targets included *FRP1* in monocytes and *CD44* and *CD74* expressed in classical M1 and M1–M2 couplings, respectively (Figure 6E). *CD74* expression was significantly higher in classical M1 than that in classical M2 (Figure 6E). Similarly, *CXCR4*, an MIF receptor, was universally signaled by each CMT type; however, its expression level was relatively higher in classical M1 (Figure 6E). In contrast, NCL was a target of both *MDK* and *PTN* and was expressed in classical M2. The immune response was not significantly affected by ligand expression, whereas *SPP1*–*CD44* signaled from classical M2 (Figure 7E), and SPP1 was significantly expressed in CMT in classical M2 (Figure 7E). A similar signal was also observed in calcification (Figure 7E).

For PCP, similar cell-cell interactions were observed between tumor cell and macrophage subtypes. Signals were transmitted by the immune response and developing epithelia, whose targets were Classical M1, M2, and M1-M2 coupling. Many signals were observed. The immune response in PCP expressed more ligands than those in ACP; particularly, *NMPT,* which was found in immune response, signaled to the integrin family receptors and was targeted to classical M1, M2, and M1-M2 coupling types (Figure 7F). In contrast, while focusing on the signals emitted from various macrophages to PCP tumor cells, SPP1, similar to ACP, primarily acted on each integrin family receptor of classical M2, targeting immune response and developing epithelial cells (Figure 7F).

Examination of cell-cell interactions in ACP and PCP revealed that while some signals were similar, functions varied greatly among tumor cell subtypes, indicating that cell-cell interactions were established between tumor and immune cells.

## Discussion

Surgical excision is the primary therapeutic intervention for craniopharyngioma; however, its anatomic location often causes neurological complications such as hypopituitarism and enuresis. Although recent clinical approaches involve using BRAF inhibitors for PCP, the molecular biological basis of these tumors are unclear, and advancements in this field of interest are imperative to develop effective treatment strategies. Therefore, we used single-cell RNA sequencing to analyze 10 craniopharyngioma cases from our institution or affiliated facilities. To the best of our knowledge, this is the first study to identify tumor cell subclusters, construct a cellular atlas for both ACP and PCP subtypes, and elucidate the underlying biological processes and cellular networks. Intercellular network analysis revealed the presence of a transcriptome that directed the biological properties of craniopharyngiomas.

Single-cell analysis helped categorize cell types within the tumor tissue, including tumor cells, immune cells, and fibroblasts. The tumor cell percentage, approximately 10–20% in all cases, aligns with prior ACP reports that indicate the presence of tumor cells with the nuclear migration of β-catenin in only a fraction of tissues.^46^ The variability in fibroblast percentages across cases may reflect differences in characteristics such as tumor hardness. The tumor tissue in all 10 cases exhibited a various cell type, suggesting that the TME was governed by various cell groups within the tissue, despite varying ratios from case to case.

Subsequently, tumor cells were classified into two major subtypes (type 1 and type 2) through subclustering based on their gene expression profiles. Few cells were categorized as ciliated cells, which were believed to originate from CCPs, a phenomenon reported in a few craniopharyngiomas but not yet formally listed in the pathology classification. These cells originated from CP5 and CP8, with CP8 diagnosed as CCP. Ciliated cells in CCP either overlap with Latke cysts or are derived from the basement membrane of Latke cysts, and a precise definition remains unestablished.^9^ Type 1 cells constituted the primary cell type in ACP tumors, whereas type 2 cells were predominant in PCP. The genetic patterns of type 1 and type 2 tumor cells were investigated, which revealed the presence of a common gene activation between these subtypes. However, *DKK3* identified in type 1 cells was reported as a Wnt signaling pathway inhibitor in the DEG analysis, suggesting an inhibitory mechanism against Wnt signaling activation within the tumor.^47^ CNV analysis revealed distinct CNVs among type 1, type 2, and mixed types, indicating genomic heterogeneity. A previous study on bulk specimens reported a low frequency of CNV in craniopharyngioma, a benign tumor,^48^ though this may have been obscured in the study using bulk specimens.

Based on the molecular characteristics of ACP and PCP tumor cells, they were classified them into three subtypes, each exhibiting distinct molecular backgrounds. Calcification is clinical feature of ACP, which displayed subcluster characteristics consistent with odontogenic epithelial origin. *LEF1* and *RUNX2* were identified as highly active transcription factors in the calcification subtype of tumor cells. *RUNX2*, a transcription factor closely related to osteoblast differentiation,^49^ was the molecular basis supporting calcification characteristics. *RELB*,^50^ a member of the nuclear factor-κB family that is closely related to immune and inflammatory responses, and *STAT3,* implicated in cell cycle progression and apoptosis inhibition,^51^ were identified as highly active transcription factors in immune and inflammatory responses,^51^ suggesting a responsive reaction of tumor cells exposed to immune response. Analyzing the tumor-immune cell interaction of ACP in tumor tissues revealed signals such as *NXA1*-*FRP1*^52^ and *MIF-CD44*/*CD74*,^53^^,^^54^ which inhibited leukocyte migration, between tumor cells and macrophages in calcification. Signals, such as *ITGB1*/*ITGA5*^55^ and NCL–PTN (expressed in classical M2), were detected in developing epithelia. In the immune response, the *SPP1*–*CD44* signal from classical M2 generally hinders the sustained proliferation of T cells.^56^^,^^57^ Collectively, these results suggest that macrophages contribute to establishing a TME suitable for tumor cell survival.

Calcification and developing epithelia receive *SPP1* signals mainly from classical M2, a molecule involved in calcification mechanisms.^58^ This result may influence calcification, as a clinical ACP feature (adenoma of the pituitary apex). Gene module and transcriptome analyses suggest that developing epithelial cells, characterized by strong epithelial formation characteristics, not only proliferate themselves but also impact integrin family receptors on macrophages, providing a conducive environment for tumor growth, such as cell adhesion. This implies that ACP is a tumor cell-derived cell line. These findings highlight the high diversity of tumor cells in ACP, as each manifest distinct cellular characteristics within the tumor tissue and establish close interactions with macrophages to shape the TME. While molecularly targeted drugs are currently unavailable for treating ACP, a comprehensive understanding of ACP tumor cell characteristics is promising for uncovering the molecular basis of individual subtypes and identifying new therapeutic targets.

PCP tumor cells are similarly classified into three subtypes and each display high immune response scores. Developing epithelial cells exhibit the second-highest proliferative capacity after the cell cycle, and the three subtypes share similarities in immune and tumor growth environments. In contrast to the immune response in ACP tumor cells, the *NAMPT-ITGB1*/*ITGA5* signaling network is detected in the immune response of PCP. It supports the maintenance of immune cell maturation and tolerance against immune environment stress.^59^

We found that different disease types in craniopharyngioma had different macrophage cluster proportions under the TME, and that they differed not only in tumor size but also in their potential to cause endocrine damage. Cell-cell interaction analysis revealed a part of the signaling exchange between tumor and immune cells.

In conclusion, we constructed comprehensive cellular atlases for ACP and PCP histotypes, characterized their molecular biology, and provided a high-resolution depiction of their inter-networks with immune cells in the TME. This study lays the groundwork for further investigations to elucidate the cellular and molecular basis of craniopharyngioma tumorigenesis and progression, with potential implications for understanding molecular mechanisms in the association between TMEs and clinical phenotypes, and for clinical treatments and drug development.

### Limitations of the study

The diversity of cell types under the TME in craniopharyngioma *in vivo* and the interaction of tumor cells with immune cells, including TAMs, remain unresolved. The molecular mechanisms that trigger different endocrine disorders are also unknown. However, the findings of this study form a basis for further studies to elucidate the cellular and molecular basis of craniopharyngioma tumorigenesis and progression, and aid clinical treatment and drug development.

## Resource availability

### Lead contact

Further information and requests for resources should be directed to the lead contact, Tomoaki Tanaka (tomoaki@restaff.chiba-u.jp).

### Materials availability

This study did not generate new unique reagents.

### Data and code availability

• Single-cell RNA-seq data have been deposited at the DDBJ Sequence Read Archive and are publicly available as of the date of publication. Accession numbers are listed in the key resources table. Microscopy data reported in this paper will be shared by the lead contact upon request.
• All code has been deposited at Zenodo and is publicly available as of the date of publication. DOIs are listed in the key resources table.
• Any additional information required to reanalyze the data reported in this paper is available from the lead contact upon request.

## Acknowledgments

We extend our sincere thanks to Yae Sano for technical support. This work was supported by grants from the 10.13039/501100001700Ministry of Education, Culture, Sports, Science and Technology (Japan): Grants-in-Aid for Scientific Research (B) #21H02974, #19H03708, and #22300325 and (C) #22K08644, #22K07205, #22K08619, #21K07145, #21K08524, #20K08397, #20K07561, #19K07635, 19K08972, #18K07439, and #18K08464; Challenging Research (Exploratory) #21K19398; Early-Career Scientists #20K17527; and Fund for the Promotion of Joint International Research (Fostering Joint International Research [A]) #19KK04071, #20KK0373, and #22KK0271, and JSPS Core-to-Core Program, (grant number: JPJSCCA20200006). T. Tanaka was supported by the Japan Society for the Promotion of Science 10.13039/501100001691KAKENHI grant JP19H03708. This work was partly supported by The 10.13039/100008732Uehara Memorial Foundation, 10.13039/501100005865Mochida Memorial Foundation for Medical and Pharmaceutical Research, The 10.13039/100007428Naito Foundation, Mitsui Life Social Welfare Foundation, Princes Takamatsu Cancer Research Fund, 10.13039/100007449Takeda Science Foundation, 10.13039/501100008667SENSHIN Medical Research Foundation, 10.13039/501100012627Japan Diabetes Foundation, 10.13039/501100008672Yamaguchi Endocrine Research Foundation, The 10.13039/100007451Cell Science Research Foundation, The 10.13039/501100003837Ichiro Kanehara Foundation for the Promotion of Medical Sciences and Medical Care, the 10.13039/501100008673Yasuda Memorial Medical Foundation, MSD Life Science Foundation, The Hamaguchi Foundation for the Advancement of Biochemistry, The 10.13039/100008273Novartis Foundation (Japan) for Promotion of Science, Kose Cosmetology Research Foundation, and the Medical Institute of Bioregulation Kyushu University Cooperative Research Project Program.

## Author contributions

Conceptualization, T.Matsuda, T.K., and T.T.; Data curation, T.Matsuda and T.K.; Formal analysis, T.Matsuda; Funding acquisition, T.T.; Investigation, T.Ma., T.K., Y.T., I.S., M.F., N.H., N.I., E.K., and T.Miki; Project administration, T.T., K.H., and Y.H.; Resources, N.F., H.N., S.Y., K.H., and Y.H.; Supervision, T.T., K.H., N.I., E.K., and Y.H.; Validation, Y.T., I.S., E.K., M.F., N.H., T.Miki, N.F., H.N., and S.Y.; Visualization, T.Matsuda, T.K., and Y.N.; Writing – original draft, T.Matsuda, T.K., and T.T.; Writing – review and editing, all authors.

## Declaration of interests

The authors declare no competing interests.

## STAR★Methods

### Key resources table

**Table:** 

REAGENT or RESOURCE	SOURCE	IDENTIFIER
**Antibodies**		

Monoclonal Mouse anti-Human CD80 Antibody	LS Bio	LS-C115599
Anti-CD163 Antibody	Abcam	ab87099; RRID: AB_11154711
Donkey anti-Mouse IgG (H+L) Highly Cross-Adsorbed Secondary Antibody, Alexa Fluor™ 594	Thermo Fisher Scientific	A-21203; RRID: AB_141633
Donkey anti-Rabbit IgG (H+L) Highly Cross- Adsorbed Secondary Antibody, Alexa Fluor™ 647	Thermo Fisher Scientific	A-31573; RRID: AB_2536183
Anti-Dkk3 antibody [EPR15611]	Abcam	ab186409
Recombinant Anti-Clusterin Antibody, Rabbit Monoclonal Antibody	Sino Biological	11297-R013
S100A8 Polyclonal Antibody	Thermo Fisher Scientific	PA5-86063; RRID: AB_2802864
Anti-beta Catenin Antibody	Abcam	ab32572
VENTANA anti-BRAF V600E (VE1) Mouse Monoclonal Primary Antibody	Roche	790-5095; RRID: AB_2833072

**Biological samples**		

Patient-derived craniopharyngioma tissue	Chiba University Hospital/Moriyama Memorial Hospital	Table S1

**Critical commercial assays**		

Chromium Next GEM Single Cell 3ʹ Reagent Kit v3.1 (Dual Index)	10x Genomics	N/A
Illumina NovaSeq 6000 Sequencing System	Illumina	N/A
Dead Cell Removal kit	Miltenyi Biotec	130-090-101
Collagenase, Type 7	Worthington	LS005332
Collagenase, Type 1	Worthington	LS004194
ACK lysing buffer	Gibco	A1049201

**Deposited data**		

Raw and analyzed data used to generate analyses shown in this manuscript: scRNASeq	This paper	JGAS : accession number JGAS000722

**Software and algorithms**		

R v4.1.2	The R Project for Statistical Computin g	https://www.r-project.org/
Cell Ranger v3.1.0	10x Genomics	https://support.10xgenomics.com/single-cell gene expression/software/pipelines/3.1/installation
Human reference gene data	10x Genomics	https://www.10xgenomics.com/jp/support/software/cell-ranger/latest/release-notes/cr-reference-release-notes#2020-a
Seurat v4.0.4	Hao et al., 2021	https://github.com/satijalab/seurat/blob/HEAD/vignettes/get_started.Rmd
inferCNV v1.10.1	Tickle et al., 2019	https://github.com/broadinstitute/infercnv
monocle3 v1.0.1	Trapnell et al., 2014	https://github.com/cole-trapnell-lab/monocle3
CellChat v1.1.3	Suoqin et al., 2021	https://github.com/sqjin/CellChat
All code	Zenodo	https://doi.org/10.5281/zenodo.12561762

### Experimental model and study participant details

Between March 2015 and November 2022, 26 patients (15 males, 11 females; median age 54 years, range 2-75 years) with suspected craniopharyngioma underwent endoscopic transsphenoidal surgery at Chiba University Hospital and Moriyama Memorial Hospital, Japan. The diagnosis was confirmed by postoperative pathological examination performed by expert pituitary tumor pathologists. Single-cell RNA sequencing was performed on samples from 10 patients following the 10× Genomics protocol, while the remaining 16 samples underwent immunohistochemistry analysis only. Data analysis was conducted using R and the Seurat package. All cases where consent was obtained, and sample analysis was possible were included in the study. The study was conducted in accordance with the Declaration of Helsinki, approved by the hospital's ethics committee (No. HS202109-06), and followed STROBE reporting guidelines. All patients provided written informed consent to participate in the study.

### Method details

#### Ethics approval

This study was conducted in accordance with the principles of the Declaration of Helsinki and in compliance with good clinical practice guidelines. This study was approved by the ethics committee at Chiba University Hospital (No. HS202109-06). Written informed consent was obtained from all participating patients.

#### Sample collection

Between March 2015 and November 2022, 26 patients were enrolled in the study. All patients were included following the STROBE reporting guidelines. These patients were suspected of having craniopharyngioma based on preoperative imaging; they underwent endoscopic transsphenoidal surgery. The diagnosis was confirmed using postoperative pathological examination. Single-cell RNA sequencing was performed on samples from 10 patients, following the 10× Genomics protocol. The remaining 16 samples were validated using immunostaining.

#### Tissue dissociation and preparation of single-cell suspension

A single-cell suspension of the craniopharyngioma samples was digested via stirring at 37°C for 90 min using collagenase I^60^ (160 μL, 100 mg/mL) and collagenase VII^61^ (400 μL, 100 mg/mL; Worthington, OH, USA) immediately after surgical removal. The suspension was then filtered through a 40-μm cell strainer (Corning, NY, USA) to remove debris and undigested large tissue fragments. After allowing the cells to settle, red blood cells were isolated using ACK lysing buffer (Thermo Fisher Scientific, MA, USA), and dead cells were removed using a dead cell removal kit (Miltenyi Biotec, Bergisch Gladbach, Germany); the resultant cell suspension contained approximately 20,000 cells per sample.

#### Single-cell RNA sequencing library preparation and sequencing

The obtained cell suspension was prepared following the specifications of 10× Genomics, using 10× Chromium Next GEM single-cell 3ʹ reagent kits and the library construction kit (10× Genomics, CA, USA). The cell suspension was loaded into the 10× Genomics Chromium Controller, creating gel beads in emulsion (GEM). After generating the GEM, the suspension was incubated at 53°C for 45 min in a thermal cycler (Veriti, Applied Biosystems, MA, USA). A barcode-linked polyA cDNA with barcodes at the 5ʹ end was produced via adding template switch oligos linked to cell barcodes and unique molecular identifiers (UMIs). The GEMs were then disrupted, and single-stranded cDNA was cleaned using DynaBeads MyOne Silane Beads (Thermo Fisher Scientific). The cDNA was amplified (98°C for 3 min, 11 cycles of 98°C for 15 s, 63°C for 20 s, and 72°C for 1 min) and the quality was assessed using Agilent TapeStation (Agilent Technologies, CA, USA). The cDNA was enzymatically fragmented, end-repaired, A-tailed, and subjected to size selection on both ends using SPRIselect beads (Beckman Coulter, CA, USA). It was then ligated with adapters provided in the kit. Sample-specific indices were introduced into each library via 14-cycle polymerase chain reaction amplification using kit-supplied indices (98°C for 45 s, 14 cycles of 98°C for 20 s, 54°C for 30 s, 72°C for 20 s, 72°C for 1 min, and holding at 4°C). Indexed libraries underwent a second round of size selection. The library was quantified and quality was assessed using Agilent TapeStation. Libraries were clustered on a NovaSeq 6000 paired-end read flow cell (Illumina, CA, USA). Sequencing was performed with reads for R1 (containing 10× barcodes and UMIs), I7 index (8 cycles; sample index), and R2 (89 cycles; transcript). The 10× Genomics Cell Ranger Single-Cell Software was used for sample demultiplexing, alignment to the human reference genome (hg19), filtering, UMI counting, single-cell 3ʹ end gene counting, and QC as per manufacturer’s instructions.

#### Data quality check, cell clustering, and cell type identification

FASTQ files were aligned using the 10× Genomics CellRanger 6.0.0. pipeline to the human GRCh38 reference transcriptome to generate gene-expression count matrices with the “exclude the introns” option. The R package Seurat (v4.0.4) was used to implement various QC procedures based on gene expression counts and the proportion of mitochondrial RNA in each sample. Initially, cells expressing > 3,000 genes (potential doublets) or < 200 genes and > 30% mitochondrial genes (indicative of low quality) were filtered. DoubletFinder (v2.0.3) was then applied to identify with pN = 0.25; doublets were removed based on the expected doublet rate derived from the loading rate. The gene count for each cell was normalized via dividing it by the total cell gene count, multiplied by a scaling factor of 10,000, and subsequently log-transformed. Variable genes were selected using the FindVariableFeatures function with default parameters. The ScaleData function was utilized to scale and center the dataset. Principal component analysis was conducted on variable genes and the number of principal components was determined based on the elbow plot results for cell clustering. Cluster markers for each cell cluster obtained through these procedures were identified using the FindAllMarkers function. Cell type identification was manually performed via comparing cluster markers with single-cell markers from PanglaoDB.^10^

After QC and doublet cell removal, the datasets were integrated using the Integrate package and clustered using a resolution of 0.5. Clusters of each cell type were annotated with representative marker genes. A resolution of 0.5 or higher was used for subclustering the following cell populations: overall cells, tumor cells, immune cells, myeloid cells, macrophages and monocytes, fibroblasts, and endothelial cells.

#### Calculating the gene expression score

Gene expression scores were calculated based on specific predefined expression programs using the AddModuleScore function in the Seurat R package (the gene sets used for scoring are listed in Table S10). The proliferation and immune response scores displayed in Figure 5G were calculated using gene sets obtained using gene module analysis.

#### Macrophage subclustering and M1/M2 scoring

For macrophage subclustering within immune cell populations, the M1 and M2 gene sets (Table S10) were used to calculate M1 and M2 scores, respectively, using the AddModuleScore function and classical M1 and M2 annotation (Figure 6B). Multivariate analysis was performed to examine endocrinological disorders and macrophage subtypes. Cells with M1 and M2 scores above 0.2, which represent the overall peak of the score distribution to define the genetic character of M1 more precisely and that of M2, were included in the analysis. Cells with both M1 and M2 scores > 0.2 were defined as “coupled” and cells with both scores < 0 were classified as “monocytes.”

#### Inferring CNVs from single-cell RNA-seq data

Single-cell CNVs were estimated using the R package inferCNV, as previously reported,^13^ via analyzing moving-averaged expression profiles across chromosomal intervals. Immune cells identified in craniopharyngioma were the reference cells. The average CNV values for these cells were subtracted from those of all other cells.

#### Gene module analysis

Gene module analysis was performed using Monocle3 (v1.0.1).^26^ Seurat was employed for clustering. Differential gene expression analysis was conducted in each subtype of ACP/PCP tumor cells. Gene modules, defined as groups of co-expressed genes, were identified using statistical methods such as weighted correlation network analysis. For the obtained gene modules, enriched biological processes were calculated.

#### Activated transcription factor analysis

Activated transcription factors were estimated using SCENIC analysis,^28^ conducted using the R package SCENIC (version 0.11.2) with default parameters. The input matrix consisted of the normalized expression matrix for the cells of interest. The output AUCell matrix was further binarized. The motif database for Homo sapiens was downloaded from cisTargetDBs (https://resources.aertslab.org/cistarget/) for reference.

#### Cell-cell interaction analysis

Cell-cell interactions were analyzed using the R package CellChat (version 1.1.3).^62^ The data obtained from subtypes of tumor cells (ACP and PCP) and immune cell subtypes, separately obtained using Seurat, were merged and loaded into CellChat. ACP and PCP cells were downsampled to 10,000 cells each for subsequent calculations.

#### Macrophage immunofluorostaining and cell counting

Macrophage immunofluorescence staining and cell counting were conducted on each sample. Following deparaffinization, antigens were retrieved using citrate buffer at pH 6, at 121°C for 5 min. After washing and blocking with 10% donkey serum at 15–25°C for 30 min, primary antibody reactions were performed for 12 h at 4°C using anti-CD80 (1:300) and anti-CD163 (1:100), as specified in the key resources table. Following another washing step, a secondary antibody reaction was performed for 1 h at room temperature (20–25°C) using CD80: AlexaFluor594 (1:500) and CD163: AlexaFluor647 (1:500). After washing, the slides were stained for nuclei using VECTASHIELD Mounting Medium (Vector Laboratories, CA, USA) with DAPI and then sealed. The prepared slides were imaged using a Zeiss LSM980 microscope (Oberkochen, Germany) and cells were counted.

#### Immunohistochemistry for marker proteins

Formalin-fixed and paraffin-embedded tumor tissues were deparaffinized with xylene and alcohol. After antigen retrieval, the sections were washed three times with phosphate buffered saline (PBS) and then blocked at room temperature for 30 min with 10% rabbit serum or goat serum. Primary antibody reactions were performed overnight at 4°C, followed by three PBS washes. An endogenous peroxidase blocking reaction (150 mL of methanol + 1.5 mL of hydrogen peroxide for 30 min) was then performed, followed by another three washes with PBS. Secondary antibody reactions (biotinylated anti-mouse or anti-rabbit antibodies) were performed at room temperature for 30 min, followed by three PBS washes. The labeling enzyme reaction (peroxidase-labeled streptavidin) was performed at room temperature for 30 min, followed by color development (DAB solution) and counterstaining with hematoxylin for 30 s. Samples were then washed and dehydrated. The antigen retrieval conditions and concentrations for each antibody are as follows:

Cytokeratin7 (Microwave 500 W for 20 min, citrate acid pH 6.0, 1:300), DKK3 (Autoclave 121°C for 5 min, citrate acid pH 6.0, 1:500), Clusterin (none, 1:100), S100A8 (none, 1:50), β-catenin (Autoclave 121°C for 5 min, 1:200). For BRAF V600E, after antigen retrieval at 98°C for 64 min, the primary antibody reaction was performed at 1:1 for 37°C for 16 min, followed by three PBS washes. An endogenous peroxidase blocking reaction (150 mL of methanol + 1.5 mL of hydrogen peroxide for 30 min) was performed. This was followed by three PBS washes, secondary antibody reaction (anti-mouse antibody horseradish peroxidase) at room temperature for 30 min, another three PBS washes, development with DAB solution, counterstaining with hematoxylin for 30 s, washing, and dehydration.

### Quantification and statistical analysis

The data were analyzed with R (version 4.1.2). Differentially expressed genes (DEGs) were identified using the FindMarkers or FindAllMarkers functions included in the Seurat package (Figures 3B, 6E, and S9E). The threshold for expression levels was set at Log2FC > 0.25 (Figures 3B, 6E, and S9E). The statistical significance of gene expression levels and scores derived using the AddModuleScore was tested using the stat_compare_means function from the ggpubr package, employing the Wilcoxon rank-sum test (Figures 1F, 2C, 4D, 5F, 6D, and S8C). The proportions of immunohistochemically positive cells were compared using the t.test function from the stats package, employing Welch’s t-test (Figure 6G).

## Supplemental information

Document S1. Figures S1–S9 and Tables S6 and S9

Table S1. Clinical information of cases analyzed by single-cell sequencing, related to Figure 1

Table S2. Clinical information of additional cases, related to Figure 6

Table S3. Differentially Expressed Genes (DEGs) of all cell clusters, related to Figure 2

Table S4. DEGs for tumor cell type1 VS type2, related to Figure 3

Table S5. DEGs of ACP subcluster, related to Figure 4

Table S7. Gene sets for module/SCENIC analysis, related to Figures 4 and 5

Table S8. DEGs of PCP subcluster, related to Figure 5

Table S10. Gene sets for scoring, related to Figures 1, 2, 5, and 6
