# Supplementary material for: Deciphering craniopharyngioma subtypes: Single-cell analysis of tumor microenvironment and immune networks
Source: iScience. 2024 Oct 1;27(11):111068. doi: 10.1016/j.isci.2024.111068 (PMC11525618; doi:10.1016/j.isci.2024.111068)
Supplement: Document S1. Figures S1–S9 and Tables S6 and S9 [file mmc1.pdf]

iScience, Volume *27*

## Supplemental information

# Deciphering craniopharyngioma subtypes: Single-cell analysis of tumor microenvironment and immune networks

**Tatsuma Matsuda, Takashi Kono, Yuki Taki, Ikki Sakuma, Masanori Fujimoto, Naoko Hashimoto, Eiryo Kawakami, Noriaki Fukuhara, Hiroshi Nishioka, Naoko Inoshita, Shozo Yamada, Yasuhiro Nakamura, Kentaro Horiguchi, Takashi Miki, Yoshinori Higuchi, and Tomoaki Tanaka**

# Supplementary Figure 1

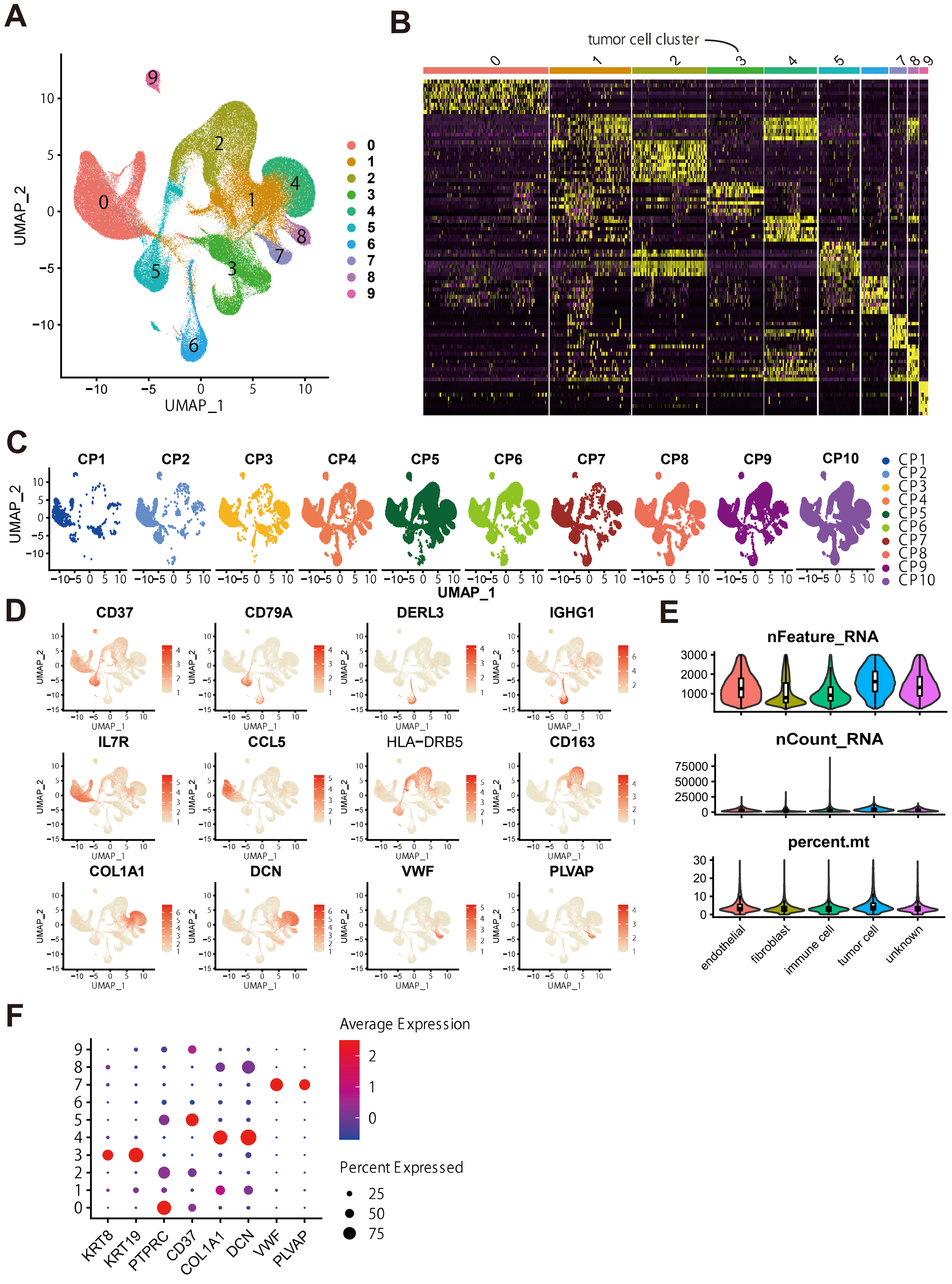

**Figure S1. | Overall clustering and annotation, related to Figure 1. A,** UMAP displaying 192,520 cells classified into 10 clusters. Clustering was performed using up to PC15, with the resolution set at 0.1. **B,** Heatmap using the top 10 DEGs based on $log_2$FC in each cluster. Cluster 3 is tumor cells. DEGs were identified using the FindAllMarkers function of the *Seurat* R package, with a threshold of $Log_2FC < 0.25$. **C,** UMAP split by individual cases. **D,** Featureplots depicting marker genes expression used for cell type annotation: *CD37*, *CD79A*, *DERL3*, *IGHG1*, *IL7R*, *CCL5*, HLA-DRB5, *CD163* for immune cells; *COL1A1*, *DCN* for fibroblasts; *VWF*, *PLVAP* for endothelial cells. **E**, Quality control results for each cell type are shown. nFeature_RNA represents the number of genes detected per cell, nCount_RNA indicates the total RNA molecule count per cell, and percent_MT shows the percentage of mitochondrial genes expressed in each cell. Each point represents an individual cell, with box plots indicating the distribution of values within each cell type. The accompanying boxplot shows the interquartile range, and the line inside represents the median. **F**, Dot plot displaying marker genes expression used for cell type annotation: *KRT8, KRT19* for tumor cells; *PTPRC*, *CD37* for immune cells; *COL1A1*, *DCN* for fibroblasts; *VWF*, *PLVAP* for endothelial cells.

## Supplementary Figure 2

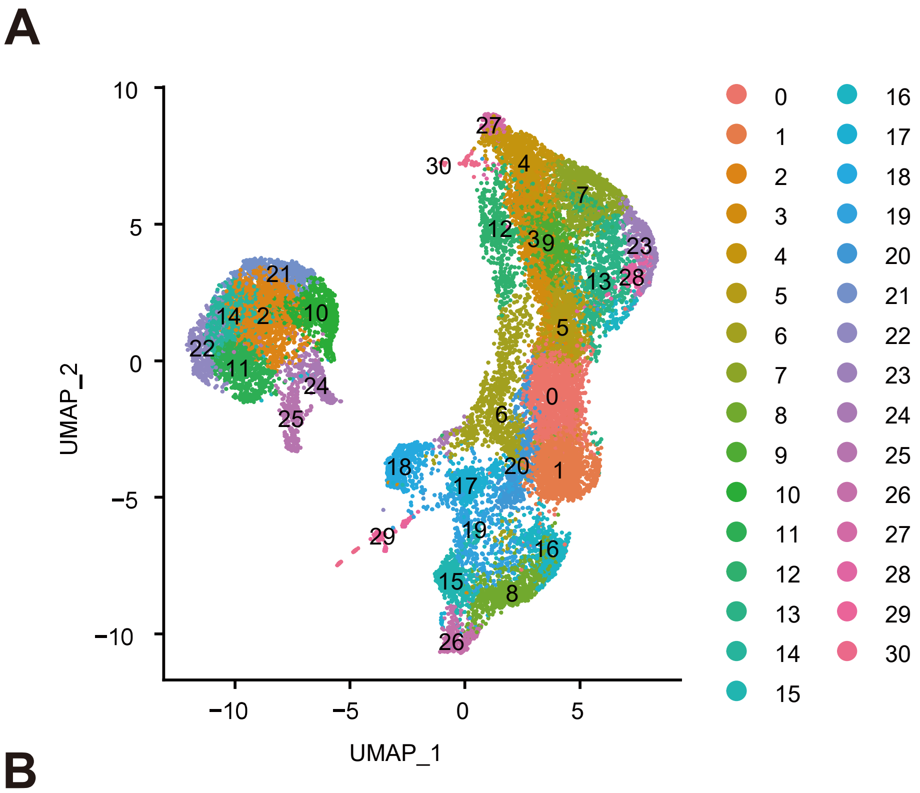


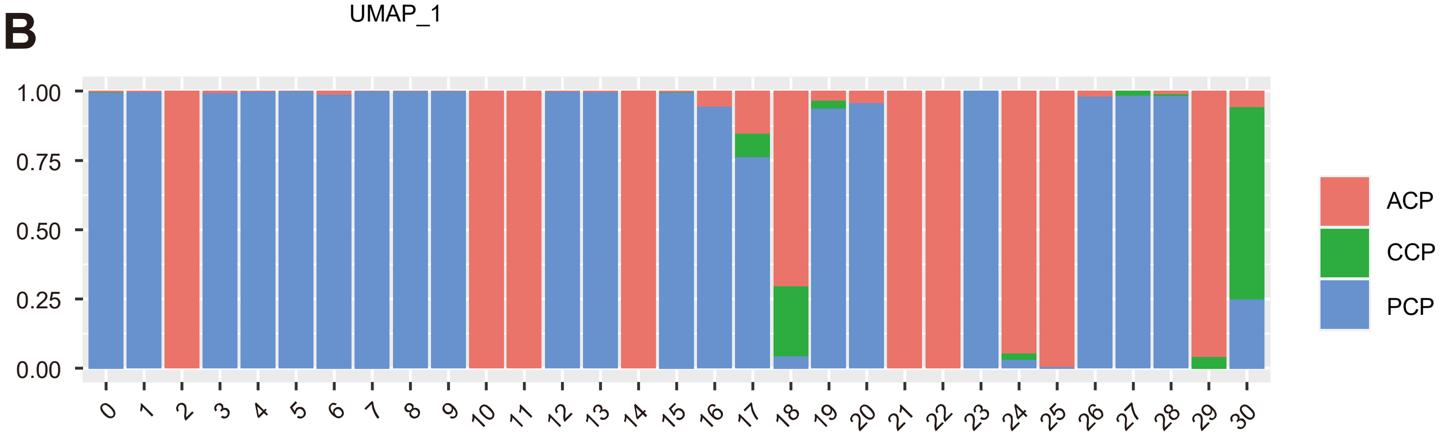

**Figure S2. | Overall clustering of tumor cells, related to Figure 2**. **A**, UMAP displaying 19,290 tumor cells, classified into 31 clusters. Clustering was performed using up to PC15, with the resolution set at 1.8. **B,** Histogram depicting the composition ratio of each pathological type on the clusters.

# Supplementary Figure 3

GO term (type1 tumor up-regulated genes)

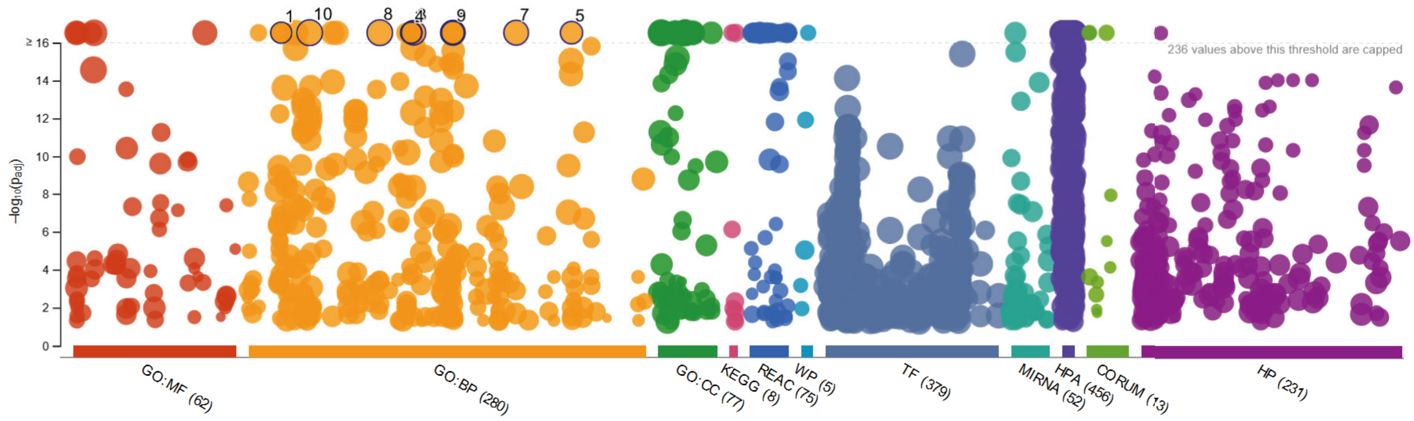


| GO:BP Term name | Term ID | $p_{adj}$ |
|---|---|---|
| peptide metabolic process | GO:0006518 | $2.861\times10^{-48}$ |
| amide biosynthetic process | GO:0043604 | $5.857\times10^{-46}$ |
| cellular nitrogen compound biosynthetic process | GO:0044271 | $4.520\times10^{-40}$ |
| amide metabolic process | GO:0043603 | $6.974\times10^{-38}$ |
| organonitrogen compound biosynthetic process | GO:1901566 | $1.368\times10^{-25}$ |
| regulation of nitrogen compound metabolic process | GO:0051171 | $3.204\times10^{-24}$ |
| regulation of primary metabolic process | GO:0080090 | $2.117\times10^{-22}$ |
| cellular nitrogen compound metabolic process | GO:0034641 | $3.192\times10^{-22}$ |
| negative regulation of nitrogen compound metabolic ... | GO:0051172 | $5.452\times10^{-21}$ |
| gene expression | GO:0010467 | $6.462\times10^{-21}$ |

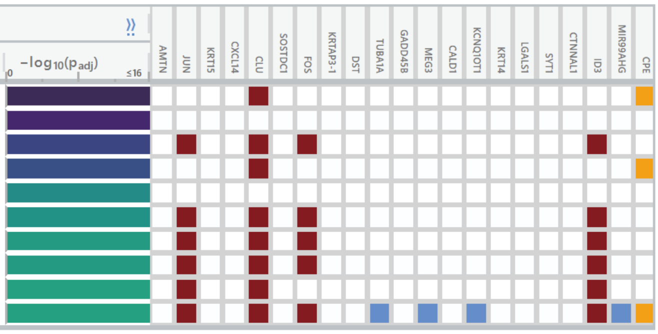


GO term (type2 tumor up-regulated genes)

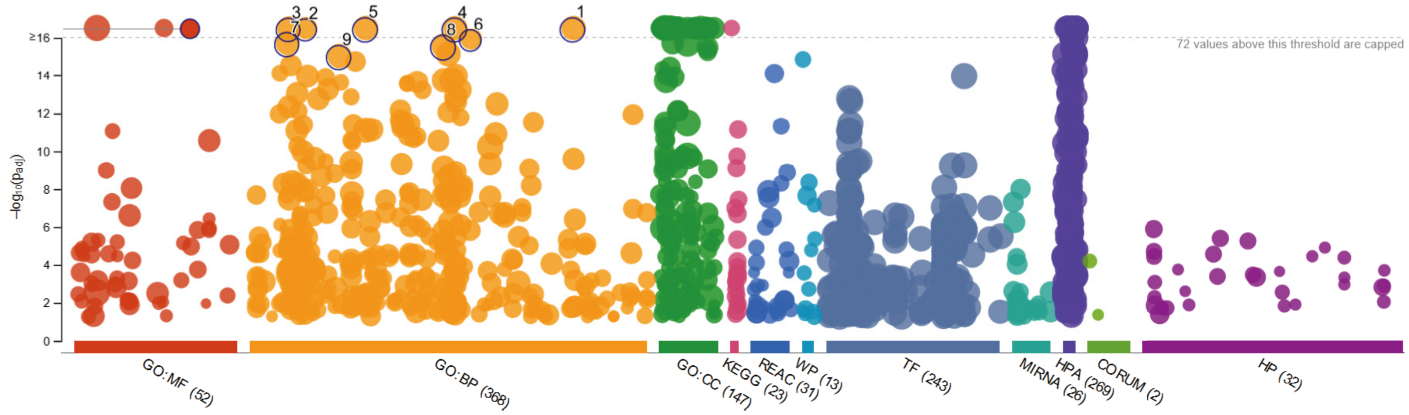


| GO:BP Term name | Term ID | $p_{adj}$ |
|---|---|---|
| organonitrogen compound metabolic process | GO:1901564 | $9.857\times10^{-24}$ |
| tissue development | GO:0009888 | $2.542\times10^{-18}$ |
| response to stress | GO:0006950 | $2.576\times10^{-17}$ |
| localization | GO:0051179 | $2.602\times10^{-17}$ |
| developmental process | GO:0032502 | $6.961\times10^{-17}$ |
| epithelium development | GO:0060429 | $1.188\times10^{-16}$ |
| transport | GO:0006810 | $1.975\times10^{-16}$ |
| positive regulation of biological process | GO:0048518 | $2.833\times10^{-16}$ |
| protein metabolic process | GO:0019538 | $9.579\times10^{-16}$ |

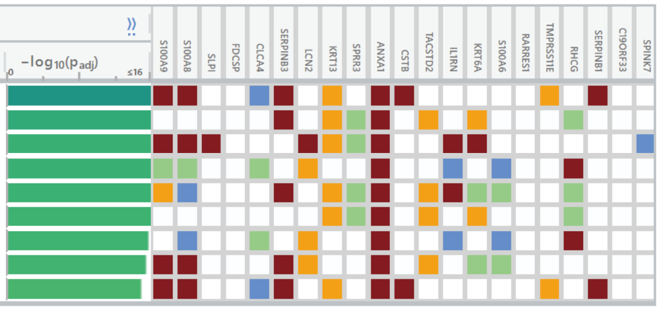

**Figure S3. | Gene Ontology (GO) analysis of DEGs between type 1 and type 2 tumor cells. Created using gProfiler, related to Figure 3.** The upper section displays GO biological processes enriched in type 1 tumor cells, and the lower section shows those enriched in type 2 tumor cells.

# Supplementary Figure 4

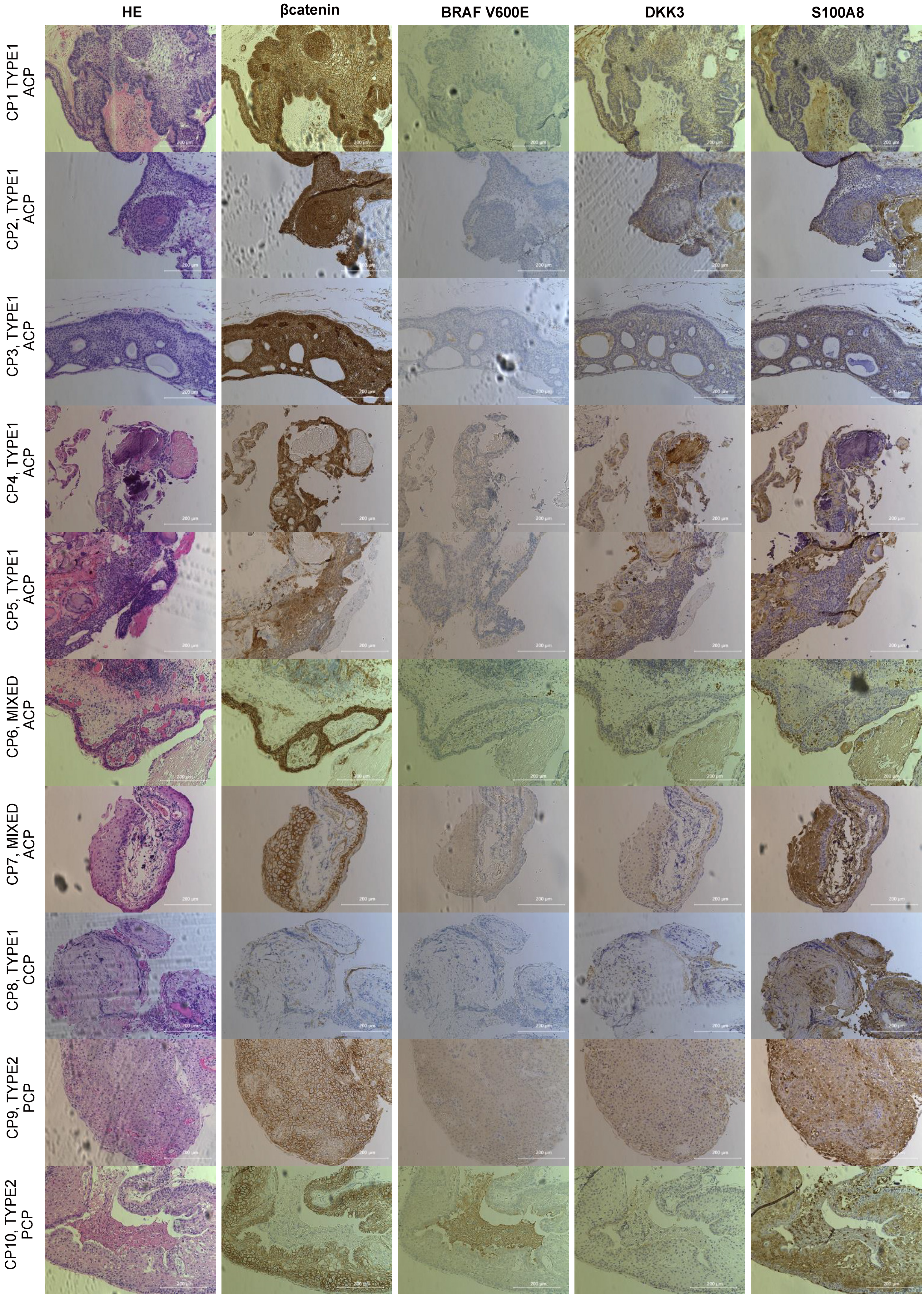

**Figure S4. | Immunostaining results across 10 cases.** Representative images of hematoxylin and eosin (H&E) staining and immunohistochemical staining for β-catenin, BRAF V600E, DKK3, and S100A8 in 10 cases. Scale bar, 200 µm.

## Supplementary Figure 5

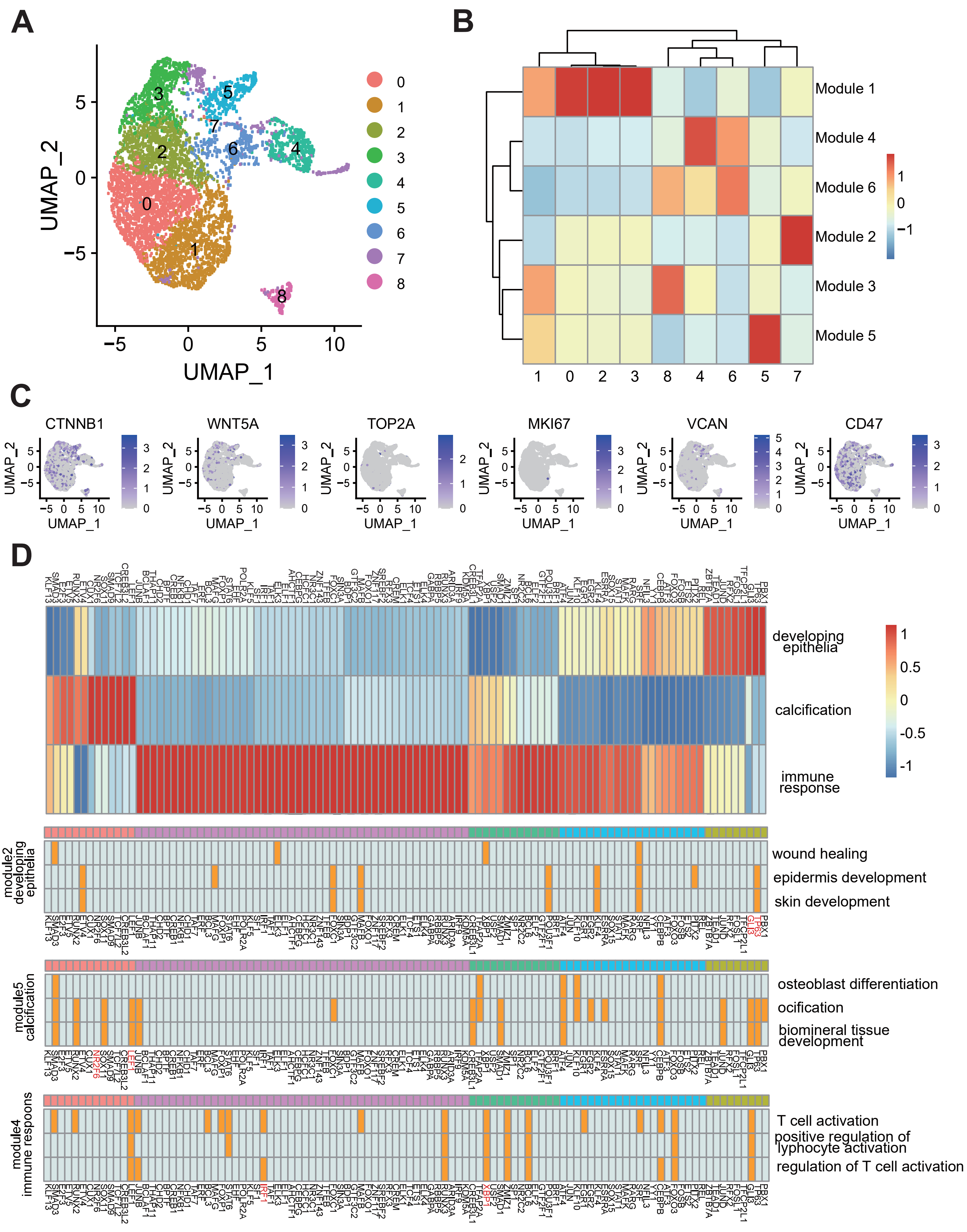

**Figure S5. | Activity of key genes and transcription factors operating in each tumor cell subtype of ACP, related to Figure 4. A**, Tumor cells in ACP, up to PC10, were classified into 9 clusters using a resolution setting of 0.5, then integrated into the following three categories based on the results of gene module analysis: developing epithelial, calcification and immune response (Figure 4A). **B**, Heatmap shows the results of module analysis for each ACP tumor cell subcluster. The modules were identified using Monocle3, with different modules active across the subclusters. The color scale represents module activity, where red indicates positive correlation (activation), blue indicates negative correlation (suppression), and white indicates intermediate activity. **C**, Featureplots showing key genes related to Wnt signal (*CTNNB1*, *WNT5A*), cell proliferation (*TOP2A*, *MKI67*), calcification (*VCAN)*, and apoptosis (*CD47*). **D**, Upper heat map shows the activity of transcription factors detected in each tumor cell subtype of ACP using SCENIC. The lower matrix shows the ontology included in module 2 (developing epithelial), module 4 (immune response), and module 5 (calcification) and the presence or absence of involvement of transcription factors.

# Supplementary Figure 6

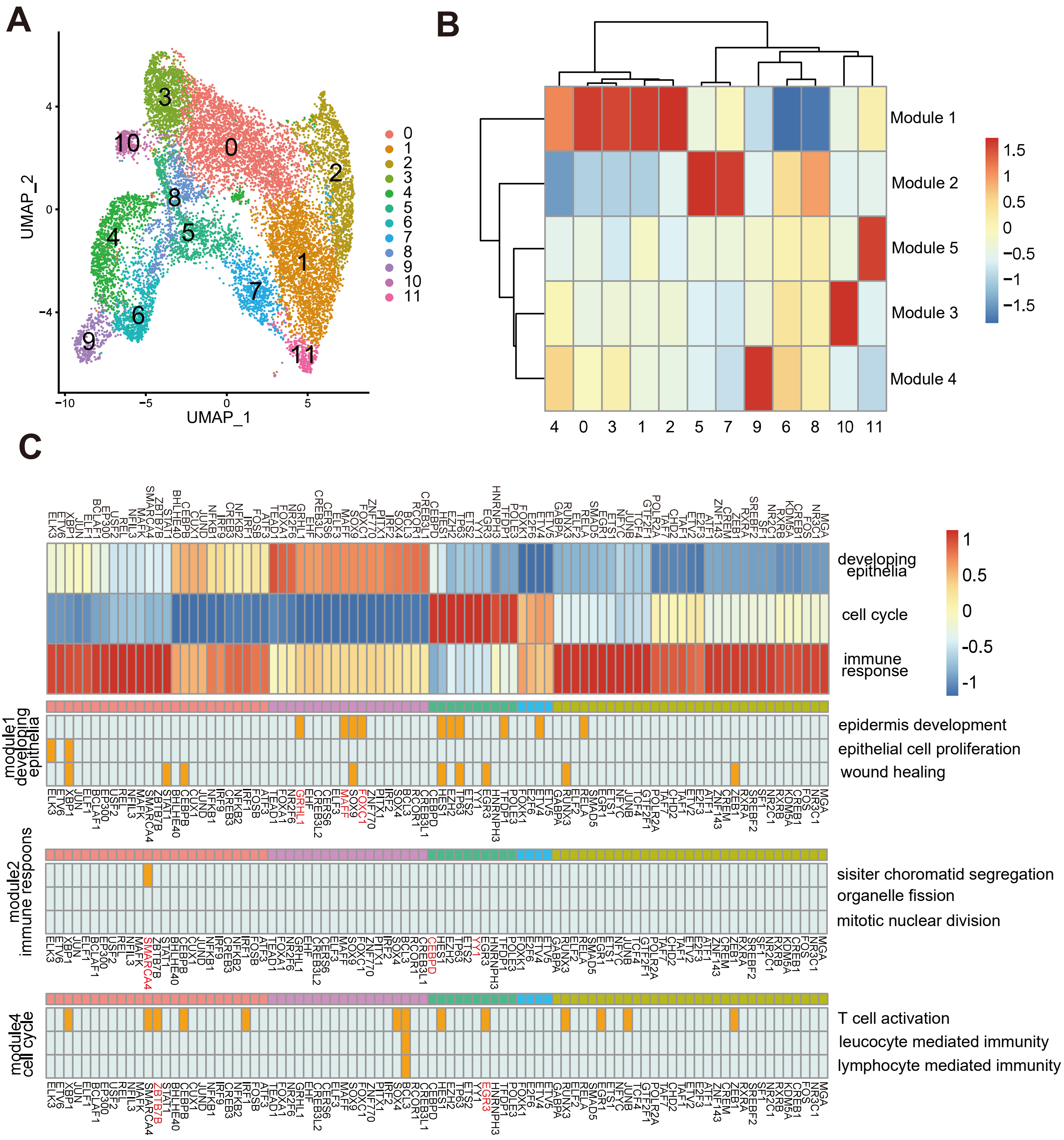

**Figure S6. | Subclustering of PCP tumor cells and SCENIC results, related to Figure 5. A,** Tumor cells in PCP, up to PC10, were classified into 12 clusters using a resolution setting of 0.5, then integrated into the following three categories based on the results of gene module analysis: developing epithelial, immune response, and cell cycle (Figure 5A). **B,** Heatmap shows the results of module analysis for each PCP tumor cell subcluster. The modules were identified using Monocle3, with different modules active across the subclusters. The color scale represents module activity, where red indicates positive correlation (activation), blue indicates negative correlation (suppression), and white indicates intermediate activity. **C,** Upper heatmap shows the activity of transcription factors detected in each tumor cell subtype of PCP using SCENIC. The lower matrix shows the gene ontology included in module 1 (developing epithelial), module 2 (immune response), and module 4 (cell cycle), as well as the presence or absence of involvement of transcription factors.

# Supplementary Figure 7

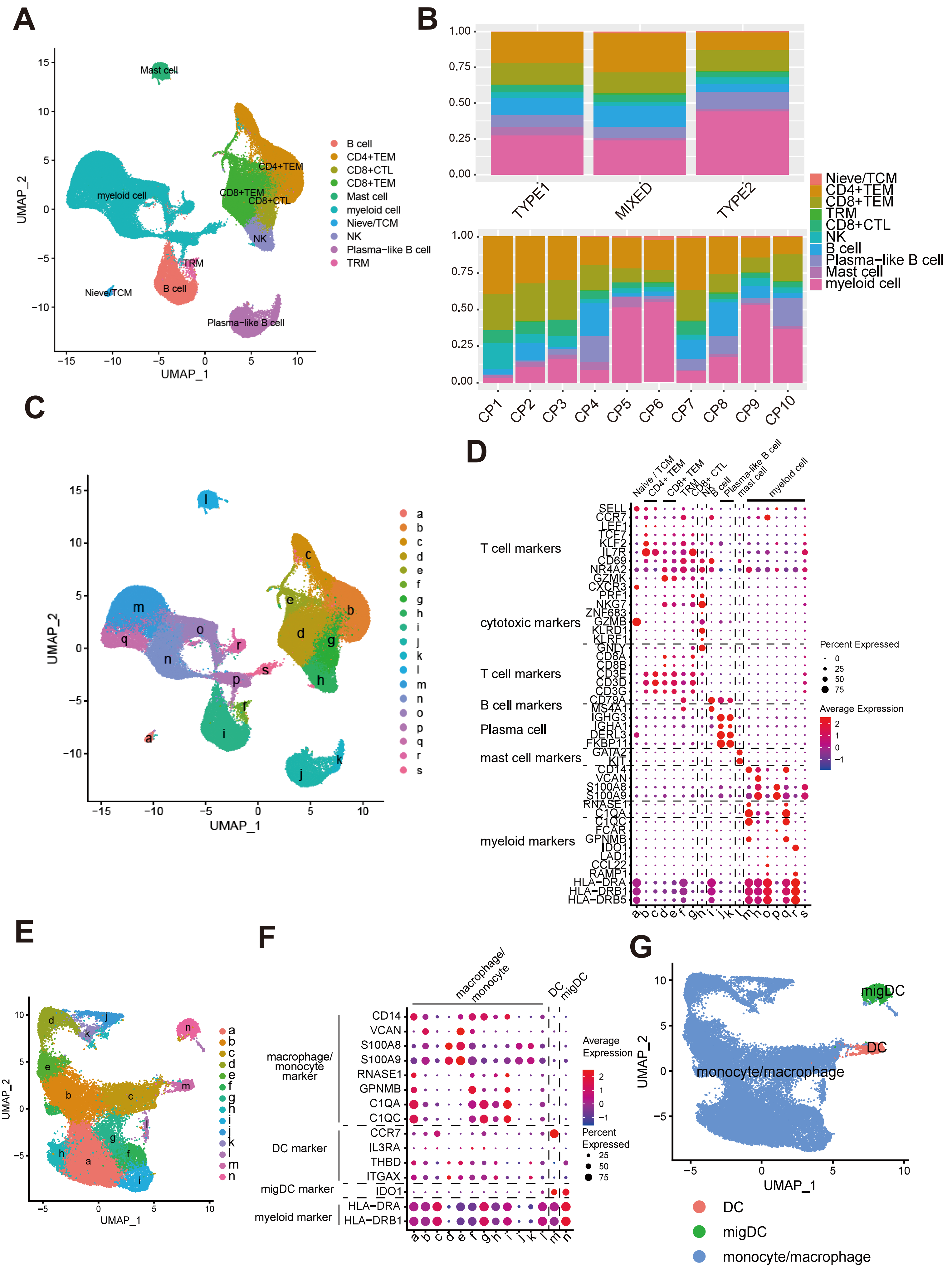

**Figure S7. | Subclustering of immune cells and cell type identification. A**, Subclustering and annotation of immune cells. A total of 105,775 immune cells were subclustered using up to PC10, and a resolution of 0.7 and their types were identified. **B**, Histogram depicting the composition ratio of each immune cell based on the type and case. **C**, 105,775 immune cells were classified into 19 clusters, and their types were identified based on the dot plot in D. **D**, Dot plot displaying the expression of marker genes of immune cell subtypes. **E**, Only myeloid cells were extracted and re-subclustered into 14 subclusters, and their types were identified based on the dot plot in F. **F**, Dot plot depicting the expression of marker genes of myeloid cell subtypes. **G**, Identified myeloid cell subclusters.

# Supplementary Figure 8

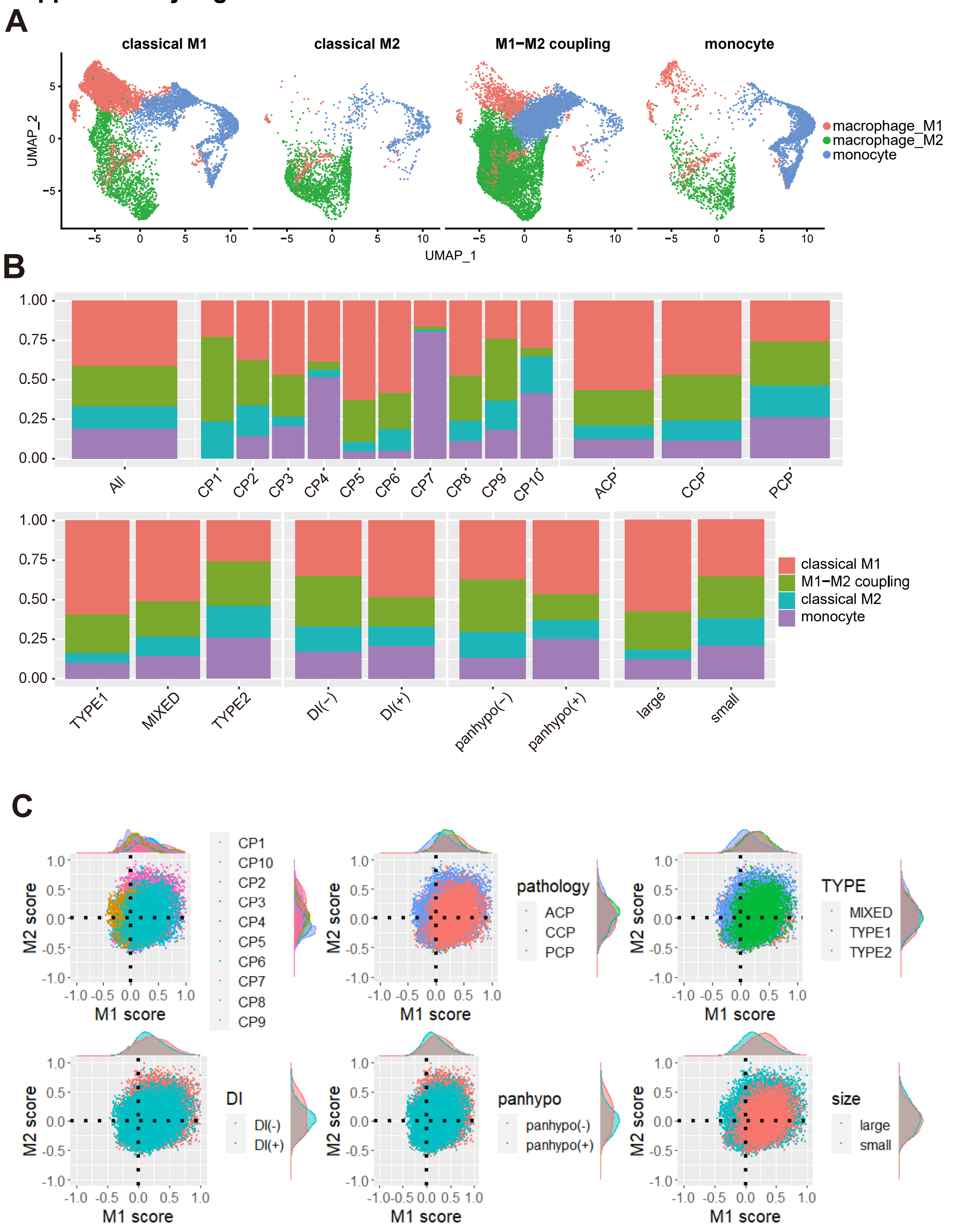

**Figure S8. | Subclustering of monocytes/macrophages, related to Figure 6. A,** Uniform Manifold Approximation and Projection (UMAP) of monocytes/macrophages, grouped by macrophage subtype, split by calculated macrophage type (CMT). **B,** Composition percentages for each type of CMT. **C,** Scatter plot of CMT scores split out by case/type/pathology/clinical significance.

# Supplementary Figure 9

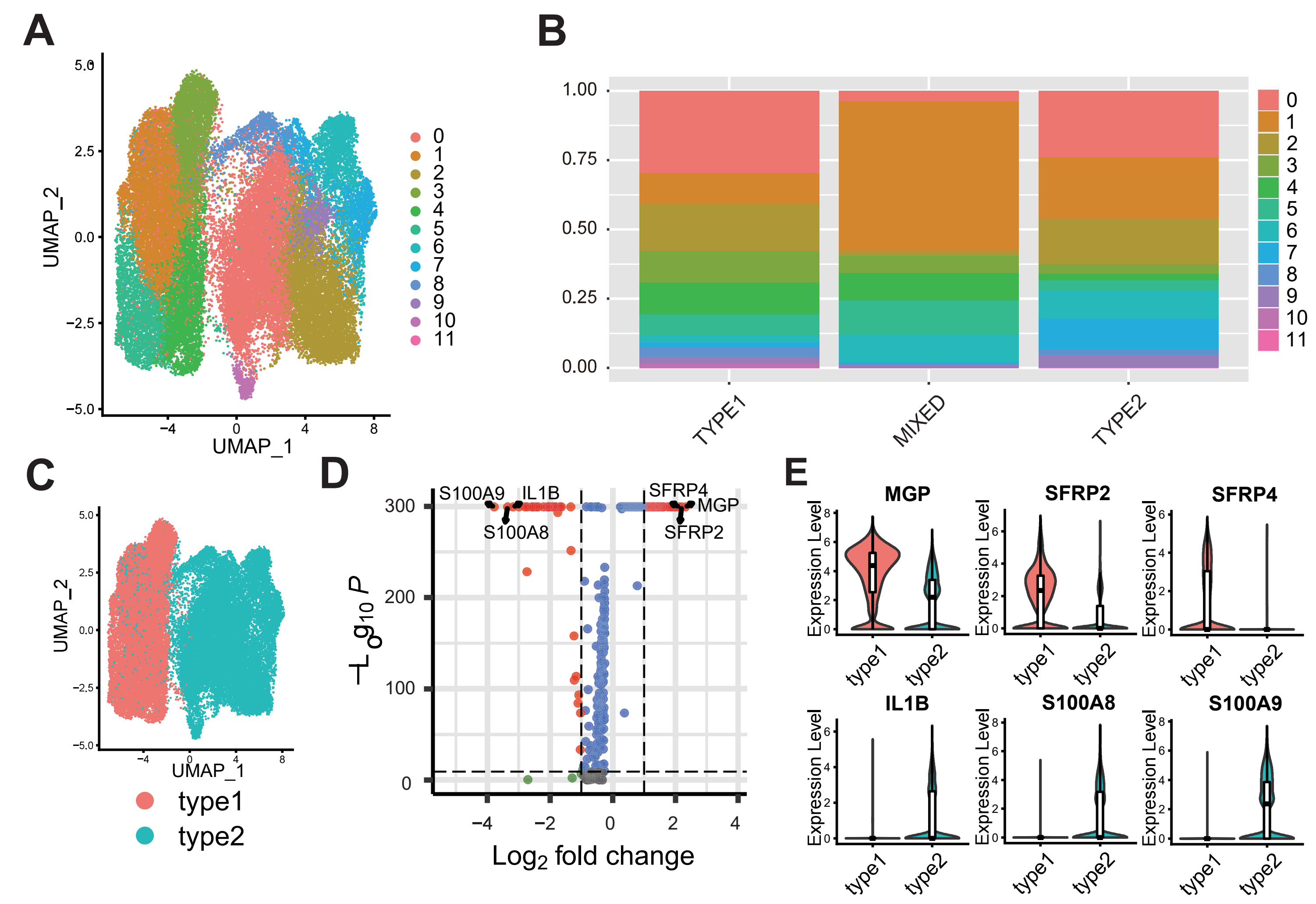


F

GO biological process (type1 fib)

| Term | P-value | Adjusted P-value |
|---|---|---|
| Extracellular Matrix Organization (GO:0030198) | 1.80E-12 | 4.79E-09 |
| Supramolecular Fiber Organization (GO:0097435) | 1.52E-10 | 2.02E-07 |
| Collagen Fibril Organization (GO:0030199) | 7.03E-10 | 5.08E-07 |
| Regulation Of Cell Migration (GO:0030334) | 7.63E-10 | 5.08E-07 |
| Negative Regulation Of Cellular Process (GO:0048523) | 7.72E-09 | 4.11E-06 |
| Regulation Of Apoptotic Process (GO:0042981) | 1.24E-08 | 5.48E-06 |
| Positive Regulation Of Cell Differentiation (GO:0045597) | 1.51E-08 | 5.76E-06 |
| Skeletal System Development (GO:0001501) | 2.15E-08 | 7.14E-06 |
| Extracellular Structure Organization (GO:0043062) | 3.76E-08 | 1.11E-05 |
| External Encapsulating Structure Organization (GO:0045229) | 4.30E-08 | 1.14E-05 |

GO biological process (type2 fib)

| Term | P-value | Adjusted P-value |
|---|---|---|
| Negative Regulation Of Apoptotic Process (GO:0043066) | 7.69E-12 | 1.54E-08 |
| Regulation Of Apoptotic Process (GO:0042981) | 1.36E-09 | 1.36E-06 |
| Negative Regulation Of Programmed Cell Death (GO:0043069) | 3.68E-09 | 2.45E-06 |
| Response To Unfolded Protein (GO:0006986) | 1.60E-08 | 7.97E-06 |
| Negative Regulation Of Gene Expression (GO:0010629) | 9.76E-08 | 3.90E-05 |
| Leukocyte Aggregation (GO:0070486) | 3.96E-07 | 1.32E-04 |
| Regulation Of Protein Modification By Small Protein Conjugation Or Removal (GO:1903320) | 4.71E-07 | 1.35E-04 |
| Positive Regulation Of Intracellular Signal Transduction (GO:1902533) | 6.92E-07 | 1.46E-04 |
| Antigen Processing And Presentation Of Exogenous Peptide Antigen (GO:0002478) | 7.18E-07 | 1.46E-04 |
| Negative Regulation Of Cysteine-Type Endopeptidase Activity Involved In Apoptotic Process (GO:0043154) | 7.29E-07 | 1.46E-04 |

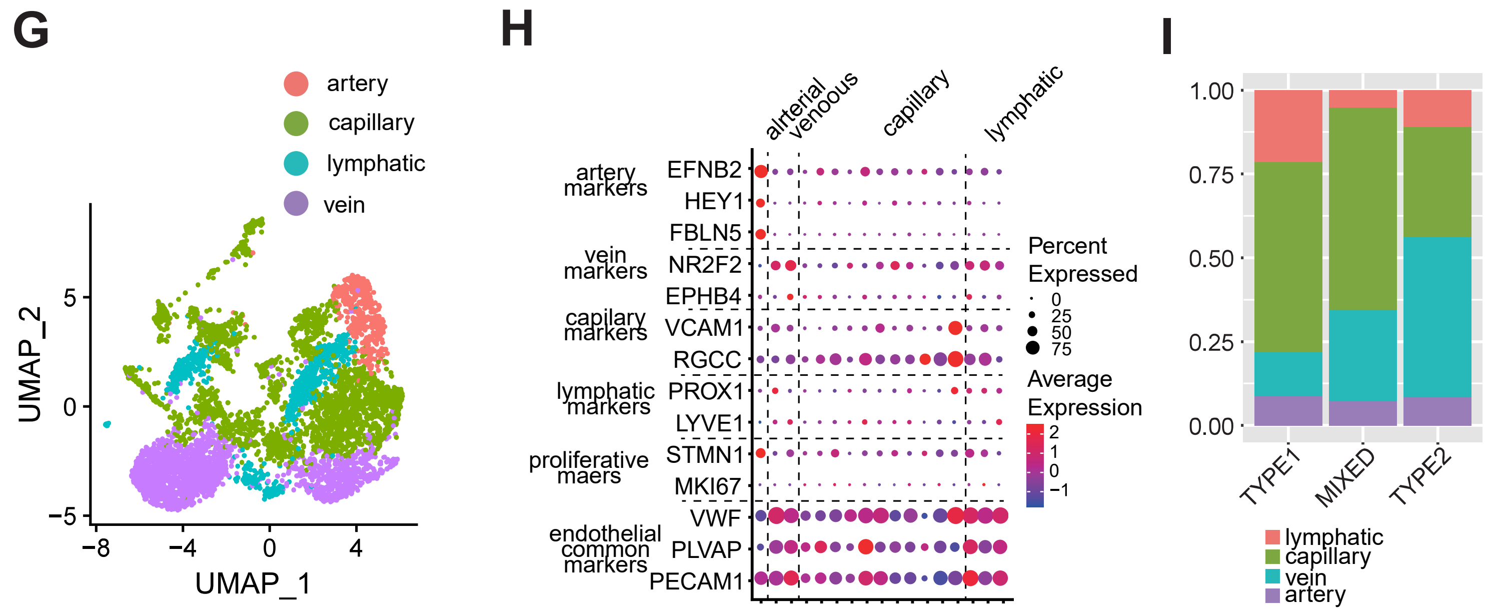

**Figure S9. | Subclustering of fibroblasts and endothelial cells. A,** A total of 55,209 fibroblasts were divided into 12 clusters using up to PC15 with a resolution setting of 0.5. **B,** Histogram displaying the composition ratio of fibroblast subtypes for each type. **C,** 55,209 fibroblasts were divided into two types. These were defined as type1_fib and type2_fib. **D,** Volcano plot of differentially expressed genes (DEGs) between type1_fib and type2_fib. Genes upregulated in type1_fib are on the right and type2_fib are on the left respectively. Genes with $Log_2FC > 1$ are indicated in red. DEGs were identified using the FindAllMarkers function in the *Seurat* R package with a threshold of $Log_2FC > 0.25$ and expression in more than 25% of cells in each type. **E,** Violin plots depicting the expression of genes specific to each type, displaying the median and interquartile range. **F,** Enriched gene ontology (GO biological process) of DEGs in type1_fib and type2_fib. **G,** A total of 7,123 endothelial cells were subclustered using up to PC15 with a resolution of 0.9. Each cluster was annotated based on the dot plot in E. **H,** Dot plot displaying the expression of marker genes of endothelial cell subtypes. **I,** Histogram depicting the composition ratio of endothelial cell subtypes for each type.

**Supplemental table 6 ACP regulon activity**

| gene | calcification | developing epithelia | immune response |
|---|---|---|---|
| ELF1 | -0.64924589 | -0.502335234 | 1.151581125 |
| ELK3 | -0.652352169 | -0.498946625 | 1.151298794 |
| BCLAF1 | -0.778687856 | -0.349055637 | 1.127743493 |
| ERF | -0.965868695 | -0.065083214 | 1.030951909 |
| NR3C1 | -0.659176643 | -0.49145651 | 1.150633153 |
| CHD2 | -0.856433974 | -0.242521887 | 1.09895586 |
| POLR2A | -0.87724752 | -0.211628184 | 1.088875704 |
| TAF7 | -0.944775257 | -0.102546963 | 1.047322221 |
| RUNX2 | -0.227595083 | -0.86658522 | 1.094180303 |
| MAFB | -0.413183315 | -0.727196146 | 1.140379461 |
| CUX1 | 1.15469932 | -0.578802323 | -0.575896997 |
| ZNF117 | -0.447222484 | -0.69833997 | 1.145562454 |
| TCF4 | -0.43232612 | -0.711102056 | 1.143428175 |
| STAT1 | -0.811272289 | -0.305966241 | 1.11723853 |
| IRF9 | -0.564135683 | -0.590464793 | 1.154600476 |
| CREB3L1 | 0.447142349 | -1.145551538 | 0.698409188 |
| XBP1 | -0.044494567 | -0.977010028 | 1.021504595 |
| FOXC1 | -0.482582048 | -0.667188984 | 1.149771032 |
| E2F3 | 0.896560831 | -1.078466108 | 0.181905277 |
| CREB3L2 | 0.873553121 | -1.09074805 | 0.217194929 |
| NR2F6 | 0.996769247 | -1.003199739 | 0.006430492 |
| SMAD9 | 1.143443879 | -0.71101326 | -0.432430618 |
| LEF1 | 1.149641529 | -0.668326197 | -0.481315332 |
| POU3F1 | -0.14636188 | -0.918753357 | 1.065115237 |
| SOX11 | 1.151883515 | -0.645750604 | -0.506132911 |
| EGR2 | -0.953961875 | -0.086461656 | 1.040423531 |
| PITX2 | -1.149496081 | 0.669585112 | 0.479910969 |
| JUNB | -0.804351472 | -0.315294844 | 1.119646315 |
| JUN | -1.147583673 | 0.462937046 | 0.684646627 |
| KLF4 | -0.841820228 | -0.263563427 | 1.105383655 |
| KLF10 | -1.089276222 | 0.21281288 | 0.876463342 |
| FOSB | -1.154518699 | 0.595005608 | 0.559513092 |
| REL | -1.115622547 | 0.299858273 | 0.815764273 |
| ATF4 | -1.057393704 | 0.126902232 | 0.930491472 |
| NFIL3 | -1.127777394 | 0.778571281 | 0.349206114 |
| CEBPB | -1.138679411 | 0.403336995 | 0.735342415 |
| ETS2 | 0.181587185 | 0.896763784 | -1.078350968 |

| | | | |
|---|---|---|---|
| YY1 | -0.742132974 | 1.137179394 | -0.395046421 |
| EGR1 | -1.046808537 | 0.945475252 | 0.101333285 |
| JUND | -0.822962127 | 1.112943823 | -0.289981696 |
| ATF3 | -0.884314902 | 1.085189616 | -0.200874714 |
| RARG | -0.532601981 | -0.620970866 | 1.153572847 |
| MAFK | -0.479321368 | -0.670113062 | 1.14943443 |
| ESRRA | -0.540074371 | -0.613840496 | 1.153914867 |
| KLF5 | -0.365479065 | -0.765848255 | 1.13132732 |
| MAFG | -0.647645699 | -0.504075868 | 1.151721567 |
| NFKB1 | -0.689784926 | -0.457072353 | 1.146857279 |
| BCL3 | -0.634752646 | -0.517977978 | 1.152730624 |
| IRF1 | -0.695565231 | -0.450428983 | 1.145994215 |
| CHD1 | -0.602983675 | -0.551331718 | 1.154315392 |
| FOXO1 | -0.367945783 | -0.763899424 | 1.131845206 |
| RUNX3 | -0.565951061 | -0.588674943 | 1.154626004 |
| ETS1 | -0.486969979 | -0.663237228 | 1.150207207 |
| CREM | -0.408929475 | -0.730726426 | 1.139655902 |
| EHF | -0.781937291 | -0.344851002 | 1.126788294 |
| USF2 | 0.586475811 | -1.154652203 | 0.568176392 |
| ELK1 | -0.404580882 | -0.734318193 | 1.138899074 |
| SMAD1 | 0.538978099 | -1.153868468 | 0.614890369 |
| ELK4 | -0.43340219 | -0.710187197 | 1.143589387 |
| ZMIZ1 | 0.024501635 | -1.012025668 | 0.987524033 |
| SREBF2 | -0.394879542 | -0.742269019 | 1.137148561 |
| ARID3A | -0.521342996 | -0.631601504 | 1.1529445 |
| AHCTF1 | -0.650623597 | -0.500833891 | 1.151457489 |
| NR2C2 | -0.266772954 | -0.839559638 | 1.106332592 |
| ELF2 | -0.164703802 | -0.90742307 | 1.072126872 |
| GTF2F1 | -0.109776902 | -0.940582178 | 1.05035908 |
| SIN3A | -0.644393722 | -0.507602842 | 1.151996565 |
| TAF1 | -0.729862857 | -0.409971893 | 1.13983475 |
| SP1 | 0.075077414 | -1.035422737 | 0.960345323 |
| GABPA | -0.446708458 | -0.698783866 | 1.145492323 |
| ETV5 | 1.043315056 | -0.950166887 | -0.093148169 |
| KLF13 | 0.531665474 | -1.15352574 | 0.621860266 |
| RFX2 | -1.009031771 | 0.99071663 | 0.01831514 |
| FOSL1 | -1.090601445 | 0.873845037 | 0.216756408 |
| ZBTB7A | -0.966031883 | 1.030817732 | -0.064785849 |
| TP63 | -0.859542742 | 1.097521104 | -0.237978362 |

| | | | |
|---|---|---|---|
| PBX1 | -0.945738435 | 1.046614901 | -0.100876466 |
| TFCP2L1 | -1.150711245 | 0.658408152 | 0.492303093 |
| TEAD1 | -1.142920908 | 0.713934484 | 0.428986424 |
| GLI3 | -0.413912195 | 1.140501769 | -0.726589574 |
| BDP1 | -0.334116288 | -0.79016426 | 1.124280547 |
| BPTF | -0.932977889 | -0.122717466 | 1.055695355 |
| KDM5A | -0.615853403 | -0.537971307 | 1.153824709 |
| FOXP1 | -0.932482643 | -0.123552786 | 1.05603543 |
| SF1 | -0.76327532 | -0.368734308 | 1.132009627 |
| TCF7L2 | 1.151832567 | -0.646352807 | -0.505479759 |
| CEBPG | -0.562219468 | -0.592350026 | 1.154569494 |
| RBBP5 | -0.37221189 | -0.76051633 | 1.13272822 |
| HCFC1 | -0.834864938 | -0.273400652 | 1.10826559 |
| THAP11 | -0.502280184 | -0.649296448 | 1.151576632 |
| RFX3 | -0.64769835 | -0.504018649 | 1.151717 |
| STAT6 | -0.908188873 | -0.163477201 | 1.071666074 |
| TFAP2A | -0.291349372 | -0.821970217 | 1.113319589 |
| SMAD3 | 0.531849167 | -1.153535054 | 0.621685888 |
| TFEB | -0.674105461 | -0.474850201 | 1.148955662 |
| CREB1 | -0.728331731 | -0.411817167 | 1.140148898 |
| GTF3C2 | -0.201569444 | -0.883861062 | 1.085430506 |
| ZNF143 | -0.607285312 | -0.546888597 | 1.154173909 |
| BRF1 | -0.082991911 | -0.955917829 | 1.03890974 |
| SRF | -1.003441729 | 0.006919367 | 0.996522362 |
| BCL6 | -0.650474105 | -0.500996921 | 1.151471026 |
| ETV4 | 0.761584585 | 0.370866999 | -1.132451584 |
| SOX15 | -1.084234505 | 0.198130837 | 0.886103668 |
| FOXO3 | -1.147946358 | 0.465971471 | 0.681974887 |

**Supplemental table 9 PCP regulon activity**

| gene | cell cycle | develiping epithelia | immune response |
|---|---|---|---|
| ZNF770 | 1.1495908 | -0.480822872 | -0.668767877 |
| TP63 | 0.6781468 | -1.148448272 | 0.470301451 |
| TCF4 | -0.868275 | -0.22508416 | 1.093359515 |
| FOXK1 | -0.607608 | -0.54655444 | 1.15416239 |
| CEBPD | 1.1418878 | -0.719500867 | -0.422386944 |
| HNRNPH3 | 0.758567 | -1.133227859 | 0.3746609 |
| YY1 | 1.0200029 | -0.978718429 | -0.04128443 |
| ETS2 | 0.9779396 | -1.020689291 | 0.042749705 |
| HES1 | -0.911222 | -0.158601032 | 1.069822744 |
| NFIL3 | -1.011205 | 0.022799602 | 0.988405247 |
| FOSB | -1.153218 | 0.525950283 | 0.627267649 |
| JUN | -1.117171 | 0.305710249 | 0.811461179 |
| JUNB | -0.704859 | -0.439643869 | 1.144502934 |
| EGR1 | -0.854642 | -0.245130042 | 1.099771983 |
| FOS | -0.722094 | -0.419295666 | 1.141389942 |
| CREM | -0.475329 | -0.673678677 | 1.149007905 |
| ETS1 | -0.472968 | -0.675780003 | 1.148748214 |
| RUNX3 | -0.388932 | -0.747101521 | 1.136033211 |
| SF1 | -0.459965 | -0.687254564 | 1.147219762 |
| NFKB2 | -1.154347 | 0.60192291 | 0.552423926 |
| XBP1 | -1.144931 | 0.442658281 | 0.702272632 |
| REL | -1.069624 | 0.158077776 | 0.911546072 |
| CREB3 | -1.150989 | 0.495382259 | 0.655606891 |
| NFKB1 | -1.154281 | 0.604105209 | 0.550175459 |
| BHLHE40 | -1.127326 | 0.78011484 | 0.347211614 |
| CUX1 | -1.096062 | 0.862651307 | 0.233410792 |
| TEAD1 | -0.77854 | 1.127786464 | -0.349246386 |
| RCOR1 | 0.0083524 | 0.995797631 | -1.004150047 |
| GRHL1 | -0.684635 | 1.14758521 | -0.462949744 |
| CERS6 | -1.079279 | 0.895121407 | 0.184157785 |
| EHF | -1.047628 | 0.944356979 | 0.103271267 |
| PITX1 | -1.041722 | 0.95226782 | 0.089453837 |
| ELF3 | -1.010868 | 0.988765766 | 0.022102059 |
| SOX9 | -0.97529 | 1.02300171 | -0.047711435 |
| IRF2 | -1.000457 | 0.999542068 | 0.000915237 |
| MAFF | -1.024419 | 0.97364654 | 0.050772592 |
| SOX4 | -0.989397 | 1.010275574 | -0.020878096 |

| | | | |
|---|---|---|---|
| FOXC1 | -0.994212 | 1.005689281 | -0.011477361 |
| JUND | -1.067794 | 0.9145048 | 0.153288939 |
| CREB3L1 | -0.939099 | 1.051417087 | -0.112318192 |
| BCL3 | -1.015466 | 0.98378075 | 0.031685385 |
| FOXA1 | -0.985227 | 1.014145416 | -0.028918123 |
| CEBPB | -1.130884 | 0.767496894 | 0.363387008 |
| IRF1 | -1.154286 | 0.603934305 | 0.550351753 |
| NR2F6 | -0.927586 | 1.059350211 | -0.131764502 |
| CREB3L2 | -1.055083 | 0.933865994 | 0.121217264 |
| ELK3 | -1.154662 | 0.58544991 | 0.569212573 |
| ATF3 | -1.154687 | 0.582235178 | 0.572451544 |
| BCLAF1 | -1.112245 | 0.287453342 | 0.82479175 |
| ELF1 | -1.133146 | 0.374259824 | 0.758886572 |
| ETV6 | -1.117804 | 0.308131107 | 0.809672697 |
| MAFK | -1.007586 | 0.01534858 | 0.992237364 |
| EP300 | -1.063275 | 0.141658322 | 0.921617155 |
| SMARCA4 | -0.988582 | -0.022457513 | 1.011039611 |
| NR3C1 | -0.749977 | -0.385374449 | 1.135350951 |
| POLR2A | -0.627819 | -0.525365915 | 1.153184497 |
| GTF2F1 | -0.946745 | -0.099127167 | 1.045871946 |
| CHD2 | -0.899517 | -0.177259198 | 1.076776543 |
| KDM5A | -0.782203 | -0.344505794 | 1.126709192 |
| TAF7 | -0.840201 | -0.265863939 | 1.106064681 |
| EGR3 | -0.107047 | -0.942169879 | 1.049217225 |
| EZH2 | 0.9425711 | -1.048927159 | 0.106356104 |
| TFDP1 | 0.8330057 | -1.109017217 | 0.276011554 |
| POLE3 | 0.883928 | -1.085394995 | 0.201466963 |
| E2F6 | -0.468793 | -0.679482106 | 1.148275561 |
| ETV4 | -0.479043 | -0.670362457 | 1.149405173 |
| TAF1 | -0.551885 | -0.60244617 | 1.154331495 |
| ATF1 | -0.850361 | -0.251329272 | 1.101689839 |
| ELF2 | -0.801402 | -0.319240204 | 1.120642614 |
| RXRA | -0.575162 | -0.579535987 | 1.154697777 |
| GABPA | -0.6197 | -0.533938345 | 1.153638436 |
| SMAD5 | -0.065104 | -0.965857208 | 1.03096135 |
| CREB1 | -0.467678 | -0.680468276 | 1.148146382 |
| SREBF2 | 0.352339 | -1.128478757 | 0.776139759 |
| USF2 | -0.874016 | -0.216498827 | 1.09051526 |
| ZBTB7B | -0.652042 | -0.499285946 | 1.151327595 |

| | | | |
|---|---|---|---|
| IRF9 | -1.154699 | 0.579013095 | 0.575685845 |
| STAT1 | -0.878401 | -0.209882847 | 1.088283629 |
| NFYC | 0.1440536 | -1.0642145 | 0.920160884 |
| NR2C1 | -0.587049 | -0.567596759 | 1.15464592 |
| ETV2 | -0.466935 | -0.681124443 | 1.148059664 |
| E2F3 | -0.618742 | -0.534944181 | 1.153686535 |
| ZEB1 | -0.469438 | -0.678911806 | 1.148349635 |
| RXRB | -0.653986 | -0.497158924 | 1.151145112 |
| RELA | -0.734224 | -0.404695465 | 1.138919238 |
| MGA | -0.75923 | -0.373828439 | 1.133058619 |
| ETV5 | -0.523822 | -0.629272856 | 1.15309439 |
| ZNF143 | -0.762142 | -0.370163989 | 1.132306343 |
